# Reference Gene Expression in Adipose-Derived Stromal Cells Undergoing Adipogenic Differentiation

**DOI:** 10.1089/ten.tec.2019.0076

**Published:** 2019-06-17

**Authors:** Carla Dessels, Michael Sean Pepper

**Affiliations:** Institute for Cellular and Molecular Medicine, Department of Immunology, and SAMRC Extramural Unit for Stem Cell Research and Therapy, Faculty of Health Sciences, University of Pretoria, Pretoria, South Africa.

**Keywords:** adipose-derived stromal cells, fetal bovine serum, human platelet lysate, adipogenesis, reference genes, cryopreservation

## Abstract

Adipose-derived stromal cells (ASCs) are becoming increasingly attractive as cellular therapy products. Their differentiation potential, the secretion of growth and differentiation factors, and the ability to cryopreserve the cells over extended periods are important features. Changes in experimental conditions result in changes in gene expression, and reverse-transcription quantitative polymerase chain reaction (RT-qPCR) has become an important tool for measuring these changes. There is, however, the potential to introduce technical bias in the process, which can be diminished through the selection of stable reference genes (RGs). Using geNorm software, in this *in vitro* study we explore the effects that adipogenic differentiation for a 21 day induction period, cryopreservation (freshly isolated ASCs or previously cryopreserved/frozen ASCs), and culture medium supplementation (fetal bovine serum vs. pooled human platelet lysate) have on the stability of 11 RGs. We found that RG stability is markedly affected by the different experimental conditions. Of the RGs assessed, *YWHAZ*, *HPRT*, *TBP*, and *ACTB* were stably expressed genes under all experimental conditions. We recommend that a panel of stable RGs should be selected before studying gene expression during adipogenesis, and that this is based on the experimental condition(s) being investigated.

**Impact Statement:**

As the use of adipose-derived stromal cells (ASCs) in clinical trials increases, so does the amount of experimental data from research groups, many of which use human ASCs to study adipogenesis in obesity. Different conditions are constantly being applied to ASCs *in vitro*, to obtain a therapeutic product for potential downstream applications. Few articles have looked at the effect of different conditions on ASC reference gene (RG) expression and stability, which was the aim of this research, as such this article will assist other researchers to make an informed decision about RG selection for gene expression studies using ASCs including those for adipogenesis.

## Background

Adipose-derived stromal cells (ASCs) are being assessed for their therapeutic potential in clinical trials.^1–3^ To be successful in ASCs' downstream applications, production needs to adhere to good manufacturing processes (GMPs).^4^ The latter can be achieved by replacing animal and chemical products with xeno-free and clinical-grade alternatives.^5^ Altering experimental conditions could alter the ASC product and this will be reflected by changes in gene expression.^6–9^ Attractive features of ASCs include the fact that they can be cryopreserved and stored for prolonged periods of time with minimal loss of their characteristics^10–12^; they remain undifferentiated during expansion^13^; and they have the potential to form adipocytes, chondrocytes, and osteocytes.^14,15^ Reverse-transcription quantitative polymerase chain reaction (RT-qPCR) is the preferred method for measuring gene expression during adipogenesis,^15–17^ and as such requires the selection and validation of a panel of internal controls or reference genes (RGs) for normalization. In this study, we examined the stability of 11 RGs used in adipogenesis studies under different experimental conditions, with the aim of defining which RGs might be the most appropriate.

## Methods

The experimental design and layout can be found in the Supplementary Figure S1.

### ASC isolation, cryopreservation, and expansion

Lipoaspirate samples were collected from four female volunteers undergoing elective liposuction. Stromal vascular fraction (SVF) containing ASCs was isolated from lipoaspirates using established protocols.^18,19^ SVF was plated at 5 × 10^5^ cells/cm^2^ in 80 cm^2^ (T80) flasks (NUNC™; Roskilde Site, Kamstrupvej, Denmark). ASCs were maintained in α-MEM containing 2% (v/v) penicillin [10,000 U/mL]–streptomycin [10,000,8 μg/mL] (p/s; GIBCO, Life Technologies™, New York, NY) and either 10% (v/v) fetal bovine serum (FBS; GIBCO, Life Technologies) or pooled human platelet lystate (pHPL) supplemented with preservative-free heparin ([2 U/mL]; Biochrom, Merck Millipore, Berlin, Germany). The pHPL was manufactured as previously described.^20,21^ At 80–90% confluence, ASCs were dissociated using tryPLE (Life Technologies) and counted. ASCs at P0 were expanded by plating 5000 cells/cm^2^ into T80 flasks and were maintained in α-MEM containing 2% (v/v) p/s and either 10% (v/v) pHPL or 10% (v/v) FBS at 37°C in 5% CO_2_. Cells remaining after seeding were cryopreserved in α-MEM containing 2% (v/v) p/s and either 10% (v/v) pHPL or 10% (v/v) FBS and 10% (v/v) dimethyl sulfoxide (DMSO) in Cryo.s™ tubes (Greiner Bio-One GmbH, Frickenhausen, Germany). The cryotubes were placed in a NALGENE^®^ Mr Frosty™ Cryo 1°C freezing container, allowing the cells to cool at a rate of 1°C per minute, and subsequently placed at −80°C overnight. ASCs cryopreserved at P0 were thawed by addition of α-MEM containing either 10% (v/v) pHPL or 10% (v/v) FBS to the cryopreservation tubes. The liquid portion containing the cellular fraction was then transferred to a conical tube (Corning, NY); this step was repeated until the ASCs were completely thawed. ASCs were then centrifuged and seeded into T80 flasks and maintained at 37°C in 5% CO_2_. Before adipogenic differentiation, the immunophenotypic surface markers (Supplementary Data) were assessed using methods previously described.^21^

### Adipogenic differentiation

At P4, ASCs were dissociated and plated for the differentiation experiment as previously described.^17^ At 80% confluence, freshly isolated ASCs expanded in FBS, previously frozen ASCs expanded in FBS, and previously frozen ASCs expanded in pHPL were induced to differentiate by replacing α-MEM supplemented medium with adipogenic induction medium consisting of DMEM (DMEM 1 × + GlutaMAX™; GIBCO, Thermo Fisher/Life Technologies™, Grand Island, NY), 2% p/s, 1 μM dexamethasone (Sigma-Aldrich Chemie, Steinheim, Germany), 0.5 mM 3-iosbutyl-methylxanthine (Sigma-Aldrich Chemie), 200 μM indomethacin (Sigma-Aldrich Chemie), and 10 μg/mL insulin (human recombinant zinc; GIBCO, Thermo Fisher/Life Technologies), and supplemented with either 10% (v/v) FBS or 5% pHPL. Both noninduced (controls) and induced ASCs were dissociated using tryPLE and their viability (Supplementary Table S1) was assessed before RNA isolation on the day of induction (day 0), and on days 1, 7, 14, and 21.

### RNA isolation, integrity and quality, and cDNA synthesis

RNA was isolated from ASCs using RNeasy Minikits (Qiagen, Hilden, Germany) according to the manufacturer's instructions, and quantified on a NanoDrop^®^ ND 1000 spectrophotometer (Thermo Fisher Scientific, Waltham, MA). RNA purity was assessed at an absorbance OD ratio of 260/280 and 260/230. Before cDNA synthesis, RNA integrity and quality were assessed using a TapeStation^®^ 2200 together with RNA ScreenTape^®^ and Sample Buffer kit according to the manufacturer's instructions (Agilent Technologies; Santa Clara, CA). RNA that had absorbance OD ratios >2 and RIN values >8 was used for downstream applications. cDNA was synthesized from 100 ng RNA using the SensiFast™ cDNA synthesis kit (Bioline, London, England) according to the manufacturer's instructions. “No RT controls” were tested and all samples displayed either no amplification or a cycle threshold (Cq) value >40.

### RGs, primer design and specificity, and amplification efficiency

Eleven RGs (Table 1) were selected based on data previously published.^8,22–24^ Primers were designed and assessed on the Integrated DNA Technologies (IDT) website. Primers were synthesized by IDT (Coralville, IA), and amplicon specificity was confirmed by the presence of single bands on agarose gel electrophoresis (Supplementary Fig. S2) and single peaks in melt curves. For each of the primers, a six-point standard curve based on a 1:2 dilution series was used and the amplification efficiency (E) and correlation coefficient (*R*^2^) were calculated (Table 1) using BioMark Real-Time PCR Analysis Software 3.1.2 (Fluidigm, South San Francisco, CA).

**Table 1.** Reference Genes, Their Functions, and Primer and qPCR Information

*Gene name and symbol*	*Gene function*	*Accession number*	*Primer pair sequences (3′–5′)*	*Amplicon length (bp)*	*Tm (°C)*	*Efficiency (E)*	*Regression coefficient (R^2^)*
Beta-actin (*ACTB*)	Cell motility, cytoskeleton structure and integrity, cytokines	NM_001101.4	F: CCAGCACAATGAAGATCAA	128	58	2.16	0.98
			R: CTCGTCATACTCCTGCTT				
β-Microglobulin (*B2M*)	Associated with the β chain of MHC I, antigen presentation	NM_004048.2	F: GTGGAGCATTCAGACTTG	138	58	2.45	0.98
			R: CTTAACTATCTTGGGCTGTG				
Glyceraldehyde 3-phosphate dehydrogenase (*GAPDH*)	Carbohydrate metabolism: oxidoreductase in glycolysis and gluconeogenesis	NM_002046.6	F: TTTGGTATCGTGGAAGGA	123	58	2.07	0.99
			R: AGGGATGATGTTCTGGAG				
Glucuronidase, beta (*GUSB)*	Hydrolysis of B-D-glucuronic acid	NM_000181.3	F: GATCGCTCACACCAAATC	132	57	2.13	0.99
			R: TCGTGATACCAAGAGTAGTAG				
Hydroxymethylbilane synthase (*HBMS*)	Heme synthesis and porphyrin metabolism	NM_001024382.1	F: GCCCTGGAGAAGAATGAA	130	58	2.13	0.98
			R: GGTGAAAGACAACAGCATC				
Hypoxanthine phosphoribosyltransferase 1 (*HPRT1*)	Purine nucleotides synthesis	NM_000194.2	F: CCTTGGTCAGGCAGTATAA	135	59	1.98	0.98
			R: GGGCATATCCTACAACAAAC				
Ribosomal protein L13a (*RPL13A*)	Structural component of 60S ribosomal subunit	NM_001270491.1	F: ATGAGGCTACGGAAACA	145	58	2.23	0.98
			R: GCAACAATGGAGGAAGG				
Ribosomal protein lateral stalk subunit P0 (*RPLP0*)	Structural component of 60S ribosomal subunit	NM_001002.3	F: ACCCTGAAGTGCTTGATA	137	58	2.19	0.98
			R: GTACCCGTTGATGATAGAATG				
Peptidylprolyl isomerase A (*PPIA*)	Protein folding through isomerization of oligopeptides	NM_001300981.1	F: CCGAAACGCCGAATATAA	116	58	1.67	0.94
			R: GGACTGTTCTTCACTCTTG				
TATA binding protein (*TBP*)	RNA polymerase II transcription factor	NM_001172085.1	F: GAGTTAAGAGTGTTGATGTAGG	130	58	2.20	0.99
			R: CCTGGGACTGGAAAGTAA				
Tyrosine 3-monooxygenase/tryptophan 5-monooxygenase activation protein, zeta (*YWHAZ*)	Signal transduction	NM_001135699.1	F: TGACATTGGGTAGCATTAAC	126	58	2.07	0.99
			R: GCACCTGACAAATAGAAAGA				

qPCR, quantitative polymerase chain reaction.

### Reverse-transcription quantitative polymerase chain reaction

High-throughput RT-qPCR was performed using Biomark^®^ 96:96 dynamic array integrated fluidic circuits (Fluidigm, South San Francisco, CA) according to manufacturer's instructions. In brief, cDNA samples were preamplified using a pool of RG primers and the following cycling conditions: 95°C for 2 min, 10 cycles of 95°C for 15 s, and 60°C for 4 min. Preamplified products were cleaned by Exonuclease I (Inqaba Biotec, Pretoria, South Africa) dilution. The following conditions were used: 95°C for 10 min, 35 cycles of 95°C for 15 s, and 60°C for 30 s. All samples were run as six technical replicates. “No template controls” and “no primer controls” were included to determine genomic amplification or the presence of primer dimers. Standard curves were run on each circuit.

### RG stability

Samples were divided into three experimental groups: (1) freshly isolated ASCs expanded in FBS (fresh FBS), (2) previously cryopreserved ASCs expanded in FBS (frozen FBS), and (3) previously cryopreserved ASCs expanded in pHPL (frozen HPL). Each group consisted of four biological replicates with nine samples each. The nine samples comprised noninduced (control) samples collected on days 0, 1, 7, 14, and 21; induced samples were collected on days 1, 7, 14, and 21. Comparisons were performed between different subsets of samples, between days, between induced and control samples, between cryopreserved and noncryopreserved ASCs, and between medium supplemented with either pHPL or FBS based on the assumptions we make in the adipogenic differentiation assay (Table 2). RG stability was determined using geNorm^25^ and comparisons were performed using the R-based NormqPCR package.^26^ The input data for geNorm required relative expression values. In this study, relative expression was calculated by converting the raw Cq values into relative expression values using the formula *E*^−ΔCq^, where ΔCq is specific RG Cq value–minimum corresponding RG Cq and either 100% efficiency was assumed (*E* = 2) or RG specific efficiencies (SEs) were used.

**Table 2.** Assumptions Made During the Adipogenic Differentiation Assay and Comparisons Used to Test the Stability of the Reference Genes Under the Different Assumptions

*Assumptions*	*Comparison*	*Example*
Does time in culture affect RG stability *Growth kinetic affects* *Comparison over time points in the same condition*	Comparison of day 0 (D0) through to day 21 (D21) in either the control or the induced ASCs for the same condition	Day 1 (D1) control frozen pHPL ASCs vs. day 7 (D7) control frozen pHPL ASCs
		Day 14 (D14) induced fresh FBS ASCs vs. day 21 (D21) induced fresh FBS ASCs
Does adipogenesis affect RG stability *Induction affects* *Comparison between induced and controls in the same condition*	Comparison of the induced and control samples on the same day for each condition	Induced day 1 (D1) frozen FBS ASCs vs. control day 1 (D1) frozen FBS ASCs
		Induced day 21 (D21) frozen pHPL ASCs vs. control day 21 (D21) frozen pHPL ASCs
Does cryopreservation affect RG stability	Comparison of the induced samples of fresh FBS ASCs and frozen FBS ASCs on the same day	Induced day 7 (D7) frozen FBS ASCs vs. induced day 7 (D7) fresh FBS ASCs
*Cryopreservation affects*		
*Comparison between fresh and frozen ASCs in the same medium*		
Does the medium affect RG stability by comparing:	Comparison between the induced samples of frozen FBS and frozen pHPL ASCs on the same day	Induced day 7 (D7) frozen FBS ASCs vs. induced day 7 (D7) frozen pHPL ASCs
*Media supplementation affects*		
*Comparison between FBS ASCs and pHPL ASCs in the same cryopreservation state*		

ASC, adipose-derived stromal cell; FBS, fetal bovine serum; p/HPL, pooled human platelet lysate; RG, reference gene.

### Statistical analysis

All data and statistical analyses were performed in RStudio (R Version 3.3.2).^27^ Descriptive statistics were calculated from the raw Cq values for all experimental conditions. To compare the means between the different control and induced groups in the same medium type for a specific RG or between medium types for a specific RG under a specific condition or at a specific time point, a Mann–Whitney U test was employed. To compare the means between days for each condition and medium type, a Kruskal–Wallis test, followed by a Dunn's *post hoc* multiple comparisons test with a Bonferroni correction, was employed. The significance level for all statistical analyses was set at α = 0.05, and a value of *p* < 0.05 was considered to be significant.

## Results

### RG expression

**FIG. 1.**
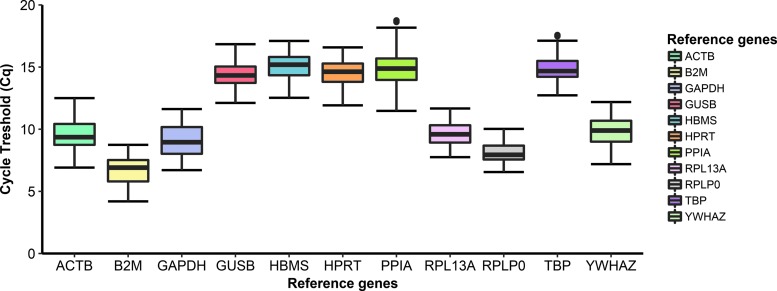
Box and whisker plots of the Cq values for the 11 RGs assessed. *Boxes* extend from the first to third quartiles with the median shown as a *solid black line* intersecting the *box*; the *whiskers* extend to the minimum and maximum values that lie within 1.5 × the IQR. *Data points* beyond the *whiskers* represent outliers. Sample size is *n* = 269 and represents all biological replicates across all experimental conditions. RG, reference gene; IQR, interquartile range; Cq, cycle threshold.

Levels of expression of the 11 RGs were determined using Cq values. The lowest average Cq value was for *B2M* (6.7 ± 1.19), and the highest value was for *HBMS* (15.1 ± 1.08). The least variable Cq values were seen for *RPLP0* (0.82) and the most variable Cq values were seen for *PPIA* (2.19). *ACTB*, *B2M*, *GAPDH*, *RPL13A*, *RPLP0*, and *YWHAZ* had the highest expression levels, whereas *GUSB*, *HBMS*, *HPRT*, *PPIA*, and *TBP* had the lowest (Fig. 1).

### Effect of adipogenic differentiation on RG expression and stability

Adipogenesis was measured using flow cytometry and fluorescence microscopy by Nile red staining as previously described.^17^ Adipogenesis was induced in all experimental groups as shown by the increase in Nile red positivity percentage (Supplementary Fig. S3) and by the appearance of lipid droplets by day 21 of adipogenic induction (Supplementary Fig. S4). We first examined the variability and stability of the 11 RGs in the control and induced samples for each time point (day 0, 1, 7, 14, and 21) for the three groups: fresh FBS, frozen FBS, and frozen HPL. Variability and significant differences were found between the Cq values in all three groups when comparing the effect and the kinetics of differentiation (Supplementary Figs. S5, S6, S7 and Supplementary Table S2).

RG stability was measured at each time point for both control and induced samples for each of the groups (Table 3), assuming either 100% efficiency (*E* = 2) or using the SEs for each of the RGs. For the fresh FBS group, when *E* = 2, *B2M*, *GAPDH*, and *YWHAZ* appeared more regularly in the higher rankings, whereas *PPIA* and *RPLP0* appeared more regularly in the lower rankings. When SE was used, *GUSB*, *TBP*, and *YWHAZ* appeared more regularly in the higher rankings, whereas *PPIA* and *RPLP0* appeared more regularly in the lower rankings. For the frozen FBS group, when *E* = 2, *GAPDH*, *HBMS*, *HPRT*, and *YWHAZ* appeared more regularly in the higher rankings, whereas *GUSB* and *RPLP0* appeared more regularly in the lower rankings. When SE was used, *B2M*, *HBMS*, *HPRT*, and *YWHAZ* appeared more regularly in the higher ranking, whereas *PPIA* and *RPLP0* appeared more regularly in the lower rankings. For the frozen HPL group, when *E* = 2, *HPRT* and *YWHAZ* appeared more regularly in the higher rankings, whereas *ACTB*, *PPIA*, and *RPLP0* appeared more regularly in the lower rankings. When the SE was used, *HBMS*, *HPRT*, and *YWHAZ* appeared more regularly in the higher rankings, whereas *ACTB*, *PPIA*, and *RPLP0* appeared more regularly in the lower rankings. To determine the overall stability for a specific group, kinetics and differentiation (controls and induced) samples were grouped together and ranked. For the fresh FBS group, the efficiency method's stability rankings were identical, where *TBP*, *YWHAZ*, *HPRT*, and *ACTB* had the greatest stability and *GUSB*, *B2M*, *RPL13A*, and *PPIA* were the least stable. In the frozen FBS group, *HPRT*, *YWHAZ*, *HBMS*, and *ACTB* were the most stable for both efficiency methods, whereas when *E* = 2, *RPL13A*, *GUSB*, *B2M*, and *RPLP0* were least stable, and when SE was used, *GUSB*, *RPL13A*, *PPIA*, and *RPLP0* were the least stable. In the frozen HPL group, *B2M*, *GUSB*, *TBP*, and *YWHAZ* displayed the highest stability and were identical for both efficiency methods, whereas *GAPDH*, *ACTB*, *RPL13A*, and *RPLP0* were the least stable when *E* = 2, and *ACTB*, *PPIA*, *RPL13A*, and *RPL13A* were the least stable for SE.

**Table 3.** Specific Efficiency and 100% Efficiency Reference Gene Stability Ranking and Values for the 11 Reference Genes in the Fresh FBS, Frozen FBS, and Frozen HPL Expanded ASCs at Each Time Point in the Control and Induced Samples

			*Fresh FBS*				*Frozen FBS*				*pHPL*			
			*100% efficiency*		*Specific efficiency*		*100% efficiency*		*Specific efficiency*		*100% efficiency*		*Specific efficiency*	
*Day*	*Differentiation*	*Rank*	*RG*	*M*	*RG*	*M*	*RG*	*M*	*RG*	*M*	*RG*	*M*	*RG*	*M*
0	Control	1	*B2M*	0.01	*GUSB*	0.01	*ACTB*	0.02	*B2M*	0.02	*HPRT*	0.03	*GAPDH*	0.02
		2	*RPL13A*	0.01	*TBP*	0.01	*PPIA*	0.02	*HPRT*	0.02	*RPLP0*	0.03	*RPLP0*	0.02
		3	*GAPDH*	0.02	*B2M*	0.02	*HPRT*	0.03	*ACTB*	0.03	*YWHAZ*	0.04	*YWHAZ*	0.04
		4	*HPRT*	0.03	*HPRT*	0.03	*HBMS*	0.04	*HBMS*	0.03	*GAPDH*	0.04	*HBMS*	0.04
		5	*YWHAZ*	0.03	*RPL13A*	0.03	*YWHAZ*	0.05	*YWHAZ*	0.04	*HBMS*	0.04	*TBP*	0.06
		6	*TBP*	0.04	*GAPDH*	0.04	*GAPDH*	0.05	*GAPDH*	0.04	*TBP*	0.05	*RPL13A*	0.06
		7	*ACTB*	0.04	*YWHAZ*	0.04	*B2M*	0.05	*RPL13A*	0.05	*RPL13A*	0.06	*HPRT*	0.06
		8	*GUSB*	0.05	*ACTB*	0.05	*RPL13A*	0.06	*TBP*	0.06	*GUSB*	0.07	*GUSB*	0.07
		9	*HBMS*	0.05	*RPLP0*	0.05	*TBP*	0.07	*GUSB*	0.06	*B2M*	0.07	*B2M*	0.08
		10	*PPIA*	0.06	*HBMS*	0.06	*GUSB*	0.08	*RPLP0*	0.06	*PPIA*	0.08	*ACTB*	0.09
		11	*RPLP0*	0.07	*PPIA*	0.08	*RPLP0*	0.08	*PPIA*	0.07	*ACTB*	0.09	*PPIA*	0.1
1	Control	1	*GAPDH*	0.04	*GAPDH*	0.04	*GAPDH*	0.02	*GAPDH*	0.01	*B2M*	0.02	*GAPDH*	0.01
		2	*YWHAZ*	0.04	*YWHAZ*	0.04	*YWHAZ*	0.02	*YWHAZ*	0.01	*HBMS*	0.02	*YWHAZ*	0.01
		3	*TBP*	0.06	*TBP*	0.07	*HPRT*	0.02	*B2M*	0.02	*RPLP0*	0.02	*B2M*	0.01
		4	*RPL13A*	0.07	*RPL13A*	0.08	*B2M*	0.02	*HPRT*	0.02	*GUSB*	0.02	*HBMS*	0.01
		5	*B2M*	0.08	*B2M*	0.08	*RPL13A*	0.03	*RPL13A*	0.02	*RPL13A*	0.02	*GUSB*	0.02
		6	*HBMS*	0.09	*HBMS*	0.1	*TBP*	0.03	*HBMS*	0.03	*GAPDH*	0.03	*RPL13A*	0.02
		7	*GUSB*	0.1	*GUSB*	0.1	*HBMS*	0.04	*ACTB*	0.03	*TBP*	0.03	*RPLP0*	0.02
		8	*ACTB*	0.1	*ACTB*	0.11	*ACTB*	0.04	*TBP*	0.03	*YWHAZ*	0.03	*TBP*	0.02
		9	*HPRT*	0.12	*HPRT*	0.12	*PPIA*	0.05	*GUSB*	0.04	*HPRT*	0.03	*HPRT*	0.03
		10	*RPLP0*	0.13	*RPLP0*	0.13	*RPLP0*	0.06	*RPLP0*	0.05	*PPIA*	0.03	*ACTB*	0.04
		11	*PPIA*	0.17	*PPIA*	0.17	*GUSB*	0.06	*PPIA*	0.06	*ACTB*	0.04	*PPIA*	0.05
1	Induced	1	*GAPDH*	0.02	*TBP*	0.01	*GUSB*	0.02	*HPRT*	0.01	*HPRT*	0.02	*HPRT*	0.02
		2	*RPL13A*	0.02	*YWHAZ*	0.01	*TBP*	0.02	*YWHAZ*	0.01	*YWHAZ*	0.02	*YWHAZ*	0.02
		3	*RPLP0*	0.03	*HPRT*	0.01	*RPL13A*	0.03	*ACTB*	0.01	*RPL13A*	0.02	*RPL13A*	0.02
		4	*GUSB*	0.04	*B2M*	0.05	*GAPDH*	0.04	*B2M*	0.02	*GUSB*	0.02	*GUSB*	0.02
		5	*B2M*	0.05	*GUSB*	0.07	*B2M*	0.04	*RPL13A*	0.04	*B2M*	0.03	*TBP*	0.03
		6	*HPRT*	0.07	*RPLP0*	0.07	*ACTB*	0.05	*TBP*	0.05	*HBMS*	0.04	*HBMS*	0.03
		7	*YWHAZ*	0.08	*GAPDH*	0.08	*YWHAZ*	0.06	*GUSB*	0.05	*TBP*	0.04	*B2M*	0.03
		8	*TBP*	0.09	*RPL13A*	0.09	*HPRT*	0.06	*GAPDH*	0.05	*GAPDH*	0.05	*ACTB*	0.04
		9	*HBMS*	0.1	*HBMS*	0.1	*HBMS*	0.06	*HBMS*	0.06	*ACTB*	0.05	*GAPDH*	0.04
		10	*ACTB*	0.11	*ACTB*	0.11	*PPIA*	0.07	*PPIA*	0.07	*PPIA*	0.06	*RPLP0*	0.05
		11	*PPIA*	0.12	*PPIA*	0.12	*RPLP0*	0.09	*RPLP0*	0.09	*RPLP0*	0.07	*PPIA*	0.07
7	Control	1	*B2M*	0.04	*GUSB*	0.02	*HBMS*	0.02	*RPL13A*	0.02	*B2M*	0.01	*B2M*	0
		2	*TBP*	0.04	*HBMS*	0.02	*YWHAZ*	0.02	*TBP*	0.02	*HBMS*	0.01	*HBMS*	0
		3	*YWHAZ*	0.05	*TBP*	0.06	*ACTB*	0.02	*GUSB*	0.03	*YWHAZ*	0.01	*YWHAZ*	0.01
		4	*ACTB*	0.06	*YWHAZ*	0.07	*HPRT*	0.03	*B2M*	0.03	*HPRT*	0.02	*HPRT*	0.02
		5	*HPRT*	0.07	*B2M*	0.08	*PPIA*	0.03	*GAPDH*	0.04	*GAPDH*	0.02	*GAPDH*	0.02
		6	*HBMS*	0.08	*ACTB*	0.09	*GAPDH*	0.04	*YWHAZ*	0.04	*RPL13A*	0.03	*RPL13A*	0.02
		7	*GUSB*	0.09	*HPRT*	0.09	*RPL13A*	0.05	*HBMS*	0.04	*ACTB*	0.03	*TBP*	0.03
		8	*GAPDH*	0.09	*RPLP0*	0.1	*TBP*	0.06	*ACTB*	0.05	*TBP*	0.04	*ACTB*	0.03
		9	*RPLP0*	0.1	*GAPDH*	0.1	*GUSB*	0.06	*HPRT*	0.05	*GUSB*	0.04	*PPIA*	0.03
		10	*RPL13A*	0.11	*RPL13A*	0.11	*B2M*	0.07	*PPIA*	0.05	*PPIA*	0.04	*GUSB*	0.04
		11	*PPIA*	0.15	*PPIA*	0.15	*RPLP0*	0.08	*RPLP0*	0.06	*RPLP0*	0.06	*RPLP0*	0.05
7	Induced	1	*ACTB*	0.02	*GUSB*	0.04	*ACTB*	0.01	*ACTB*	0.01	*HPRT*	0.02	*HPRT*	0.01
		2	*YWHAZ*	0.02	*TBP*	0.04	*HBMS*	0.01	*HPRT*	0.01	*YWHAZ*	0.02	*YWHAZ*	0.01
		3	*TBP*	0.03	*B2M*	0.04	*HPRT*	0.01	*HBMS*	0.01	*B2M*	0.03	*B2M*	0.02
		4	*PPIA*	0.04	*ACTB*	0.05	*YWHAZ*	0.02	*YWHAZ*	0.02	*GUSB*	0.03	*TBP*	0.03
		5	*GAPDH*	0.04	*YWHAZ*	0.05	*PPIA*	0.02	*B2M*	0.02	*TBP*	0.04	*HBMS*	0.04
		6	*GUSB*	0.05	*GAPDH*	0.06	*B2M*	0.02	*GUSB*	0.03	*HBMS*	0.05	*GUSB*	0.04
		7	*B2M*	0.06	*HPRT*	0.07	*TBP*	0.03	*GAPDH*	0.03	*ACTB*	0.06	*ACTB*	0.05
		8	*HPRT*	0.07	*PPIA*	0.08	*GAPDH*	0.03	*TBP*	0.03	*PPIA*	0.06	*GAPDH*	0.06
		9	*HBMS*	0.08	*RPLP0*	0.09	*GUSB*	0.04	*RPL13A*	0.04	*GAPDH*	0.07	*RPL13A*	0.08
		10	*RPLP0*	0.09	*HBMS*	0.09	*RPL13A*	0.04	*PPIA*	0.05	*RPL13A*	0.09	*PPIA*	0.1
		11	*RPL13A*	0.1	*RPL13A*	0.11	*RPLP0*	0.06	*RPLP0*	0.06	*RPLP0*	0.12	*RPLP0*	0.13
14	Control	1	*HBMS*	0.03	*HBMS*	0.03	*HBMS*	0.01	*HBMS*	0.01	*GUSB*	0.01	*GUSB*	0.01
		2	*YWHAZ*	0.03	*YWHAZ*	0.03	*HPRT*	0.01	*HPRT*	0.01	*HPRT*	0.01	*HPRT*	0.01
		3	*RPL13A*	0.03	*RPL13A*	0.03	*GAPDH*	0.01	*GAPDH*	0.01	*YWHAZ*	0.01	*YWHAZ*	0.01
		4	*ACTB*	0.04	*ACTB*	0.04	*YWHAZ*	0.02	*YWHAZ*	0.02	*HBMS*	0.02	*HBMS*	0.01
		5	*HPRT*	0.04	*TBP*	0.04	*ACTB*	0.02	*ACTB*	0.02	*B2M*	0.02	*B2M*	0.02
		6	*GAPDH*	0.04	*HPRT*	0.05	*B2M*	0.03	*B2M*	0.03	*RPL13A*	0.02	*RPL13A*	0.02
		7	*TBP*	0.05	*GAPDH*	0.05	*RPL13A*	0.04	*TBP*	0.03	*PPIA*	0.02	*PPIA*	0.02
		8	*RPLP0*	0.05	*RPLP0*	0.05	*TBP*	0.04	*RPL13A*	0.04	*GAPDH*	0.02	*GAPDH*	0.03
		9	*GUSB*	0.06	*GUSB*	0.06	*GUSB*	0.05	*GUSB*	0.04	*TBP*	0.03	*ACTB*	0.03
		10	*B2M*	0.08	*B2M*	0.08	*RPLP0*	0.06	*RPLP0*	0.05	*ACTB*	0.04	*TBP*	0.04
		11	*PPIA*	0.09	*PPIA*	0.1	*PPIA*	0.06	*PPIA*	0.07	*RPLP0*	0.04	*RPLP0*	0.04
14	Induced	1	*GUSB*	0.02	*TBP*	0.02	*GAPDH*	0.01	*GAPDH*	0.01	*ACTB*	0.01	*ACTB*	0.01
		2	*YWHAZ*	0.02	*YWHAZ*	0.02	*HBMS*	0.01	*HBMS*	0.01	*GAPDH*	0.01	*YWHAZ*	0.01
		3	*HPRT*	0.03	*GUSB*	0.02	*YWHAZ*	0.01	*YWHAZ*	0.01	*GUSB*	0.01	*HBMS*	0.01
		4	*TBP*	0.03	*GAPDH*	0.03	*B2M*	0.01	*B2M*	0.01	*YWHAZ*	0.02	*GAPDH*	0.01
		5	*B2M*	0.03	*HPRT*	0.04	*HPRT*	0.01	*HPRT*	0.01	*HBMS*	0.02	*GUSB*	0.02
		6	*GAPDH*	0.04	*RPLP0*	0.04	*ACTB*	0.01	*ACTB*	0.01	*B2M*	0.02	*B2M*	0.02
		7	*HBMS*	0.04	*B2M*	0.04	*GUSB*	0.02	*GUSB*	0.02	*HPRT*	0.03	*HPRT*	0.03
		8	*RPLP0*	0.05	*HBMS*	0.05	*PPIA*	0.02	*PPIA*	0.02	*TBP*	0.04	*TBP*	0.04
		9	*ACTB*	0.06	*ACTB*	0.05	*TBP*	0.03	*TBP*	0.03	*PPIA*	0.05	*PPIA*	0.06
		10	*PPIA*	0.08	*PPIA*	0.08	*RPL13A*	0.03	*RPL13A*	0.03	*RPL13A*	0.07	*RPL13A*	0.07
		11	*RPL13A*	0.11	*RPL13A*	0.11	*RPLP0*	0.05	*RPLP0*	0.05	*RPLP0*	0.1	*RPLP0*	0.1
21	Control	1	*GAPDH*	0.01	*ACTB*	0.02	*HBMS*	0.01	*ACTB*	0.01	*HBMS*	0.01	*HBMS*	0.01
		2	*HBMS*	0.01	*HPRT*	0.02	*HPRT*	0.01	*HBMS*	0.01	*HPRT*	0.01	*HPRT*	0.01
		3	*HPRT*	0.02	*HBMS*	0.02	*GAPDH*	0.01	*PPIA*	0.01	*B2M*	0.02	*B2M*	0.02
		4	*ACTB*	0.02	*GAPDH*	0.02	*YWHAZ*	0.02	*HPRT*	0.02	*PPIA*	0.02	*TBP*	0.02
		5	*RPLP0*	0.04	*YWHAZ*	0.04	*ACTB*	0.02	*YWHAZ*	0.02	*GAPDH*	0.03	*YWHAZ*	0.03
		6	*RPL13A*	0.04	*RPL13A*	0.05	*B2M*	0.03	*TBP*	0.03	*YWHAZ*	0.03	*GAPDH*	0.03
		7	*YWHAZ*	0.05	*RPLP0*	0.06	*RPL13A*	0.04	*GAPDH*	0.03	*GUSB*	0.04	*RPL13A*	0.03
		8	*B2M*	0.06	*TBP*	0.07	*TBP*	0.04	*B2M*	0.04	*RPL13A*	0.04	*GUSB*	0.03
		9	*TBP*	0.07	*B2M*	0.08	*GUSB*	0.05	*RPL13A*	0.04	*TBP*	0.04	*ACTB*	0.04
		10	*GUSB*	0.08	*GUSB*	0.09	*RPLP0*	0.06	*GUSB*	0.05	*ACTB*	0.04	*RPLP0*	0.04
		11	*PPIA*	0.12	*PPIA*	0.14	*PPIA*	0.06	*RPLP0*	0.06	*RPLP0*	0.05	*PPIA*	0.05
21	Induced	1	*GUSB*	0.02	*ACTB*	0.02	*GAPDH*	0.01	*GAPDH*	0	*B2M*	0.01	*HBMS*	0
		2	*YWHAZ*	0.02	*GUSB*	0.02	*HBMS*	0.01	*YWHAZ*	0	*YWHAZ*	0.01	*YWHAZ*	0
		3	*HBMS*	0.02	*HBMS*	0.03	*YWHAZ*	0.01	*HPRT*	0.01	*ACTB*	0.01	*B2M*	0.01
		4	*ACTB*	0.03	*YWHAZ*	0.03	*B2M*	0.01	*B2M*	0.01	*HBMS*	0.01	*ACTB*	0.01
		5	*TBP*	0.03	*TBP*	0.03	*HPRT*	0.01	*ACTB*	0.02	*GAPDH*	0.01	*PPIA*	0.01
		6	*B2M*	0.04	*B2M*	0.04	*ACTB*	0.01	*HBMS*	0.02	*HPRT*	0.02	*GAPDH*	0.01
		7	*HPRT*	0.04	*HPRT*	0.04	*GUSB*	0.02	*GUSB*	0.02	*PPIA*	0.02	*HPRT*	0.02
		8	*GAPDH*	0.06	*GAPDH*	0.06	*PPIA*	0.02	*PPIA*	0.03	*TBP*	0.02	*TBP*	0.02
		9	*RPL13A*	0.07	*RPLP0*	0.07	*TBP*	0.03	*TBP*	0.03	*GUSB*	0.03	*GUSB*	0.03
		10	*RPLP0*	0.08	*RPL13A*	0.08	*RPL13A*	0.03	*RPL13A*	0.04	*RPL13A*	0.05	*RPL13A*	0.04
		11	*PPIA*	0.1	*PPIA*	0.11	*RPLP0*	0.05	*RPLP0*	0.05	*RPLP0*	0.07	*RPLP0*	0.07
All		1	*TBP*	0.07	*TBP*	0.07	*HPRT*	0.03	*HPRT*	0.03	*B2M*	0.05	*B2M*	0.05
		2	*YWHAZ*	0.07	*YWHAZ*	0.07	*YWHAZ*	0.03	*YWHAZ*	0.03	*GUSB*	0.05	*GUSB*	0.05
		3	*HPRT*	0.08	*HPRT*	0.08	*HBMS*	0.04	*HBMS*	0.04	*TBP*	0.06	*TBP*	0.05
		4	*ACTB*	0.09	*ACTB*	0.09	*ACTB*	0.04	*ACTB*	0.04	*YWHAZ*	0.07	*YWHAZ*	0.06
		5	*GAPDH*	0.1	*GAPDH*	0.1	*PPIA*	0.05	*GAPDH*	0.04	*HBMS*	0.07	*HBMS*	0.07
		6	*HBMS*	0.1	*HBMS*	0.11	*GAPDH*	0.05	*TBP*	0.05	*PPIA*	0.07	*HPRT*	0.07
		7	*RPLP0*	0.12	*RPLP0*	0.12	*TBP*	0.06	*B2M*	0.05	*HPRT*	0.08	*GAPDH*	0.08
		8	*GUSB*	0.13	*GUSB*	0.13	*RPL13A*	0.06	*GUSB*	0.06	*GAPDH*	0.08	*ACTB*	0.08
		9	*B2M*	0.15	*B2M*	0.15	*GUSB*	0.07	*RPL13A*	0.06	*ACTB*	0.08	*PPIA*	0.09
		10	*RPL13A*	0.16	*RPL13A*	0.17	*B2M*	0.08	*PPIA*	0.07	*RPL13A*	0.1	*RPL13A*	0.1
		11	*PPIA*	0.18	*PPIA*	0.18	*RPLP0*	0.09	*RPLP0*	0.08	*RPLP0*	0.11	*RPLP0*	0.11

### Effect of cryopreservation on RG expression and stability

**FIG. 2.**
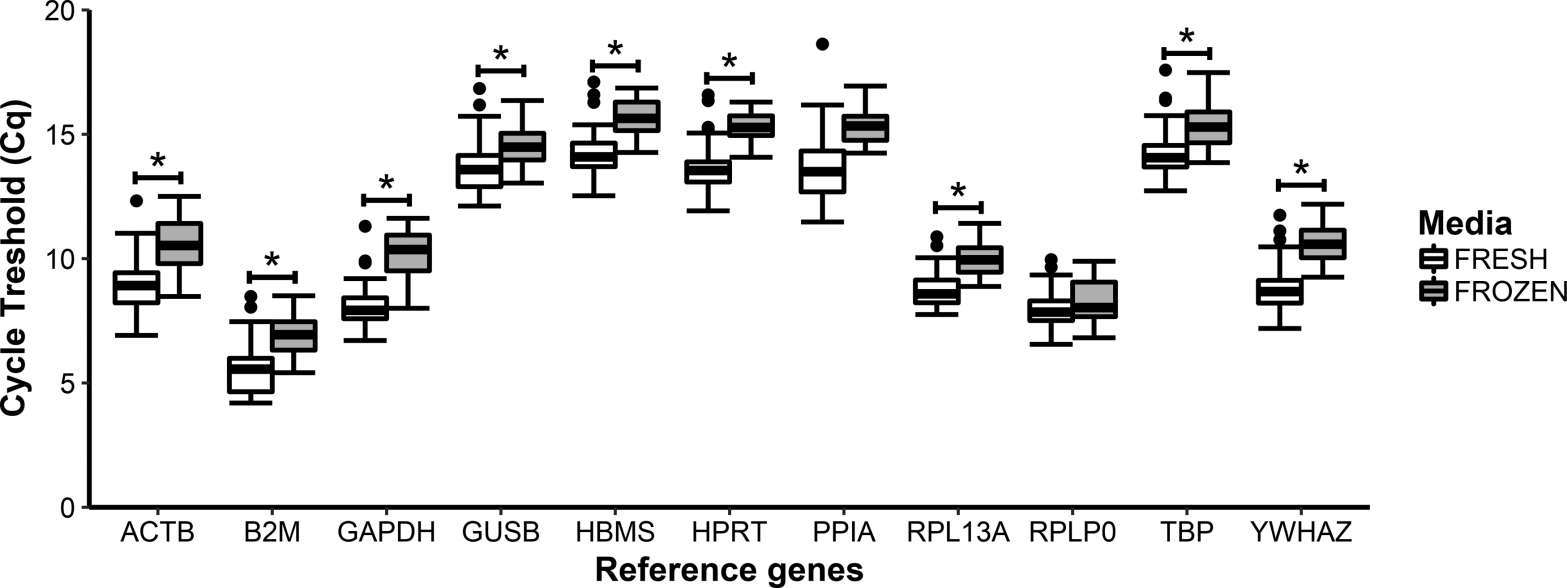
Box and whisker plots of the Cq values for the 11 RGs assessed in freshly isolated ASCs expanded in FBS and previously cryopreserved ASCs expanded in FBS. *Boxes* extend from the first to third quartiles with the median shown as the *solid black line* intersecting the *box*; *whiskers* extend to the minimum and maximum values that lie within 1.5 × the IQR. *Data points* beyond the *whiskers* represent outliers. The sample size is *n* = 36 and represents four biological replicates at nine time points at different differentiation states (day 0, days 1 control and induced, day 7 control and indicated, day 14 control and induced and day 21 control and induced). *Statistical significance *p* < 0.05. ASC, adipose-derived stromal cell; FBS, fetal bovine serum.

We next examined the effect of cryopreservation on RG expression and stability by grouping all the samples together for the fresh FBS and frozen FBS groups. When compared, the frozen FBS group had significantly higher Cq values for all RGs, except for *PPIA* and *RPLP0* (Fig. 2).

RG stability was measured for each time point in both control and induced samples for each of the groups (Table 4; Fresh FBS vs. Frozen FBS) using both efficiency methods. When *E* = 2, *HPRT*, *HBMS*, and *YWHAZ* appeared more regularly in the higher rankings, whereas *GUSB*, *B2M*, and *RPLP0* appeared more regularly in the lower rankings. When the SE was used, *HBMS*, *HPRT*, and *YWHAZ* appeared more regularly in the higher rankings, whereas *RPLP0* and *PPIA* appeared more regularly in the lower rankings. When all samples were combined and *E* = 2, *HPRT*, *YWHAZ*, *ACTB*, and *TBP* were the most stable and *B2M*, *PPIA*, *RPLP0*, and *RPL13A* were the least stable. When the SE was used, *HPRT*, *YWHAZ*, *TBP*, and *ACTB* were the most stable, whereas *RPLP0*, *B2M*, *RPL13A*, and *PPIA* were the least stable.

**Table 4.** Specific and 100% Efficiency Reference Gene Stability Rankings and Values for the 11 Reference Genes in the Fresh FBS and Frozen FBS Groups and in the Frozen FBS and Frozen HPL Groups at Each Time Point, in Induced and Control Samples

			*Fresh FBS vs. frozen FBS*				*Frozen FBS vs. frozen HPL*			
			*100% efficiency*		*Specific efficiency*		*100% efficiency*		*Specific efficiency*	
*Day*	*Differentiation*	*Rank*	*RG*	*M*	*RG*	*M*	*RG*	*M*	*RG*	*M*
0	Control	1	*GAPDH*	0.03	*ACTB*	0.03	*GAPDH*	0.02	*GUSB*	0.04
		2	*YWHAZ*	0.03	*HPRT*	0.03	*YWHAZ*	0.02	*RPL13A*	0.04
		3	*HPRT*	0.04	*YWHAZ*	0.04	*HBMS*	0.04	*B2M*	0.05
		4	*B2M*	0.05	*HBMS*	0.05	*PPIA*	0.07	*TBP*	0.06
		5	*ACTB*	0.05	*GAPDH*	0.05	*TBP*	0.09	*RPLP0*	0.06
		6	*HBMS*	0.06	*B2M*	0.06	*RPLP0*	0.1	*GAPDH*	0.09
		7	*TBP*	0.07	*TBP*	0.06	*RPL13A*	0.1	*YWHAZ*	0.1
		8	*RPL13A*	0.07	*GUSB*	0.07	*B2M*	0.11	*HBMS*	0.1
		9	*GUSB*	0.08	*RPL13A*	0.07	*GUSB*	0.11	*PPIA*	0.11
		10	*PPIA*	0.08	*RPLP0*	0.08	*HPRT*	0.12	*HPRT*	0.12
		11	*RPLP0*	0.1	*PPIA*	0.1	*ACTB*	0.13	*ACTB*	0.13
1	Control	1	*GAPDH*	0.07	*GAPDH*	0.07	*HPRT*	0.02	*HPRT*	0.03
		2	*TBP*	0.07	*HPRT*	0.07	*YWHAZ*	0.02	*YWHAZ*	0.03
		3	*HBMS*	0.07	*HBMS*	0.07	*RPL13A*	0.03	*RPL13A*	0.03
		4	*HPRT*	0.08	*TBP*	0.08	*B2M*	0.04	*B2M*	0.04
		5	*YWHAZ*	0.09	*YWHAZ*	0.09	*HBMS*	0.04	*HBMS*	0.04
		6	*ACTB*	0.09	*ACTB*	0.1	*PPIA*	0.05	*TBP*	0.05
		7	*B2M*	0.11	*B2M*	0.11	*TBP*	0.05	*RPLP0*	0.05
		8	*RPL13A*	0.12	*RPL13A*	0.12	*GAPDH*	0.06	*GUSB*	0.05
		9	*GUSB*	0.13	*GUSB*	0.13	*ACTB*	0.07	*GAPDH*	0.06
		10	*RPLP0*	0.14	*RPLP0*	0.15	*RPLP0*	0.07	*ACTB*	0.07
		11	*PPIA*	0.16	*PPIA*	0.17	*GUSB*	0.08	*PPIA*	0.07
1	Induced	1	*HPRT*	0.08	*HPRT*	0.03	*HPRT*	0.02	*HPRT*	0.01
		2	*YWHAZ*	0.08	*YWHAZ*	0.03	*YWHAZ*	0.02	*YWHAZ*	0.01
		3	*TBP*	0.09	*B2M*	0.05	*ACTB*	0.03	*ACTB*	0.03
		4	*ACTB*	0.1	*GUSB*	0.06	*HBMS*	0.04	*HBMS*	0.03
		5	*HBMS*	0.12	*TBP*	0.07	*RPL13A*	0.04	*RPL13A*	0.04
		6	*GAPDH*	0.12	*ACTB*	0.08	*B2M*	0.05	*B2M*	0.04
		7	*B2M*	0.13	*RPL13A*	0.1	*TBP*	0.06	*TBP*	0.05
		8	*GUSB*	0.14	*GAPDH*	0.11	*PPIA*	0.07	*GUSB*	0.06
		9	*RPL13A*	0.14	*HBMS*	0.12	*GUSB*	0.08	*PPIA*	0.07
		10	*RPLP0*	0.15	*PPIA*	0.12	*GAPDH*	0.09	*GAPDH*	0.09
		11	*PPIA*	0.17	*RPLP0*	0.13	*RPLP0*	0.1	*RPLP0*	0.1
7	Control	1	*GUSB*	0.05	*GUSB*	0.05	*HBMS*	0.03	*HBMS*	0.03
		2	*RPL13A*	0.05	*HBMS*	0.05	*YWHAZ*	0.03	*HPRT*	0.03
		3	*HBMS*	0.06	*TBP*	0.06	*HPRT*	0.03	*YWHAZ*	0.03
		4	*TBP*	0.07	*YWHAZ*	0.06	*PPIA*	0.04	*GAPDH*	0.04
		5	*YWHAZ*	0.08	*HPRT*	0.07	*GAPDH*	0.05	*TBP*	0.04
		6	*GAPDH*	0.08	*GAPDH*	0.08	*RPL13A*	0.06	*RPL13A*	0.05
		7	*HPRT*	0.09	*B2M*	0.08	*TBP*	0.06	*PPIA*	0.05
		8	*B2M*	0.09	*RPL13A*	0.09	*ACTB*	0.07	*GUSB*	0.06
		9	*ACTB*	0.1	*ACTB*	0.09	*GUSB*	0.08	*B2M*	0.06
		10	*RPLP0*	0.11	*RPLP0*	0.1	*B2M*	0.08	*ACTB*	0.07
		11	*PPIA*	0.13	*PPIA*	0.12	*RPLP0*	0.09	*RPLP0*	0.08
7	Induced	1	*ACTB*	0.02	*ACTB*	0.02	*HPRT*	0.03	*B2M*	0.03
		2	*YWHAZ*	0.02	*YWHAZ*	0.02	*YWHAZ*	0.03	*GUSB*	0.03
		3	*PPIA*	0.03	*GAPDH*	0.04	*HBMS*	0.04	*YWHAZ*	0.04
		4	*GAPDH*	0.04	*HPRT*	0.05	*B2M*	0.04	*HBMS*	0.04
		5	*HPRT*	0.04	*HBMS*	0.05	*GUSB*	0.05	*HPRT*	0.05
		6	*TBP*	0.05	*B2M*	0.06	*PPIA*	0.05	*TBP*	0.05
		7	*B2M*	0.06	*TBP*	0.07	*TBP*	0.06	*ACTB*	0.06
		8	*HBMS*	0.06	*PPIA*	0.08	*ACTB*	0.06	*GAPDH*	0.07
		9	*GUSB*	0.07	*GUSB*	0.08	*GAPDH*	0.07	*PPIA*	0.08
		10	*RPL13A*	0.08	*RPL13A*	0.09	*RPL13A*	0.08	*RPL13A*	0.09
		11	*RPLP0*	0.09	*RPLP0*	0.1	*RPLP0*	0.1	*RPLP0*	0.11
14	Control	1	*HBMS*	0.03	*ACTB*	0.04	*HBMS*	0.02	*HBMS*	0.01
		2	*YWHAZ*	0.03	*YWHAZ*	0.04	*HPRT*	0.02	*HPRT*	0.01
		3	*ACTB*	0.04	*HBMS*	0.04	*YWHAZ*	0.02	*YWHAZ*	0.02
		4	*HPRT*	0.04	*HPRT*	0.04	*GAPDH*	0.03	*GAPDH*	0.03
		5	*RPL13A*	0.04	*TBP*	0.04	*PPIA*	0.04	*ACTB*	0.04
		6	*TBP*	0.05	*RPL13A*	0.05	*ACTB*	0.05	*TBP*	0.04
		7	*GAPDH*	0.06	*GUSB*	0.06	*TBP*	0.05	*RPL13A*	0.05
		8	*GUSB*	0.07	*GAPDH*	0.06	*RPL13A*	0.06	*RPLP0*	0.06
		9	*B2M*	0.08	*RPLP0*	0.07	*RPLP0*	0.06	*GUSB*	0.06
		10	*PPIA*	0.09	*B2M*	0.09	*GUSB*	0.07	*B2M*	0.07
		11	*RPLP0*	0.09	*PPIA*	0.1	*B2M*	0.08	*PPIA*	0.08
14	Induced	1	*B2M*	0.02	*B2M*	0.03	*HBMS*	0.01	*HBMS*	0.01
		2	*HPRT*	0.02	*YWHAZ*	0.03	*HPRT*	0.01	*YWHAZ*	0.01
		3	*YWHAZ*	0.02	*GUSB*	0.03	*YWHAZ*	0.03	*GAPDH*	0.02
		4	*GUSB*	0.03	*TBP*	0.04	*GAPDH*	0.03	*ACTB*	0.02
		5	*TBP*	0.03	*HPRT*	0.04	*PPIA*	0.03	*GUSB*	0.03
		6	*GAPDH*	0.04	*GAPDH*	0.04	*ACTB*	0.03	*HPRT*	0.03
		7	*HBMS*	0.04	*HBMS*	0.05	*TBP*	0.04	*TBP*	0.04
		8	*ACTB*	0.05	*ACTB*	0.05	*RPL13A*	0.04	*B2M*	0.04
		9	*RPLP0*	0.06	*RPLP0*	0.06	*RPLP0*	0.05	*PPIA*	0.05
		10	*PPIA*	0.07	*PPIA*	0.08	*GUSB*	0.06	*RPL13A*	0.06
		11	*RPL13A*	0.09	*RPL13A*	0.1	*B2M*	0.09	*RPLP0*	0.08
21	Control	1	*ACTB*	0.02	*ACTB*	0.03	*HBMS*	0.01	*HBMS*	0.01
		2	*HBMS*	0.02	*HBMS*	0.03	*HPRT*	0.01	*HPRT*	0.01
		3	*HPRT*	0.03	*HPRT*	0.04	*PPIA*	0.03	*YWHAZ*	0.03
		4	*YWHAZ*	0.05	*YWHAZ*	0.05	*YWHAZ*	0.04	*TBP*	0.03
		5	*GAPDH*	0.06	*TBP*	0.06	*GAPDH*	0.04	*GAPDH*	0.04
		6	*TBP*	0.07	*GAPDH*	0.07	*TBP*	0.05	*ACTB*	0.04
		7	*RPL13A*	0.08	*RPL13A*	0.08	*RPL13A*	0.05	*PPIA*	0.05
		8	*RPLP0*	0.08	*RPLP0*	0.09	*ACTB*	0.06	*RPL13A*	0.06
		9	*GUSB*	0.1	*GUSB*	0.1	*RPLP0*	0.07	*RPLP0*	0.07
		10	*B2M*	0.11	*B2M*	0.12	*GUSB*	0.08	*GUSB*	0.07
		11	*PPIA*	0.13	*PPIA*	0.14	*B2M*	0.09	*B2M*	0.08
21	Induced	1	*ACTB*	0.03	*ACTB*	0.03	*B2M*	0.02	*B2M*	0.02
		2	*HBMS*	0.03	*HBMS*	0.03	*HBMS*	0.02	*YWHAZ*	0.02
		3	*GUSB*	0.03	*B2M*	0.03	*YWHAZ*	0.02	*HBMS*	0.02
		4	*YWHAZ*	0.03	*GUSB*	0.03	*HPRT*	0.02	*HPRT*	0.02
		5	*B2M*	0.04	*YWHAZ*	0.04	*GAPDH*	0.03	*PPIA*	0.02
		6	*HPRT*	0.04	*TBP*	0.04	*PPIA*	0.03	*GAPDH*	0.03
		7	*TBP*	0.05	*HPRT*	0.05	*ACTB*	0.03	*ACTB*	0.03
		8	*GAPDH*	0.06	*GAPDH*	0.06	*TBP*	0.03	*TBP*	0.03
		9	*PPIA*	0.07	*RPLP0*	0.07	*GUSB*	0.04	*GUSB*	0.04
		10	*RPLP0*	0.09	*RPL13A*	0.09	*RPL13A*	0.05	*RPL13A*	0.04
		11	*RPL13A*	0.1	*PPIA*	0.11	*RPLP0*	0.07	*RPLP0*	0.06
All		1	*HPRT*	0.06	*HPRT*	0.06	*HBMS*	0.04	*HPRT*	0.05
		2	*YWHAZ*	0.06	*YWHAZ*	0.06	*HPRT*	0.04	*YWHAZ*	0.05
		3	*ACTB*	0.07	*TBP*	0.07	*PPIA*	0.05	*HBMS*	0.05
		4	*TBP*	0.08	*ACTB*	0.07	*GAPDH*	0.06	*TBP*	0.06
		5	*GAPDH*	0.08	*HBMS*	0.08	*YWHAZ*	0.07	*GAPDH*	0.07
		6	*HBMS*	0.09	*GAPDH*	0.09	*ACTB*	0.07	*ACTB*	0.07
		7	*GUSB*	0.1	*GUSB*	0.1	*TBP*	0.08	*PPIA*	0.08
		8	*B2M*	0.12	*RPLP0*	0.12	*RPL13A*	0.09	*B2M*	0.09
		9	*PPIA*	0.13	*B2M*	0.13	*GUSB*	0.09	*GUSB*	0.09
		10	*RPLP0*	0.14	*RPL13A*	0.14	*B2M*	0.1	*RPL13A*	0.1
		11	*RPL13A*	0.15	*PPIA*	0.15	*RPLP0*	0.11	*RPLP0*	0.11

### Effect of medium on RG expression and stability

**FIG. 3.**
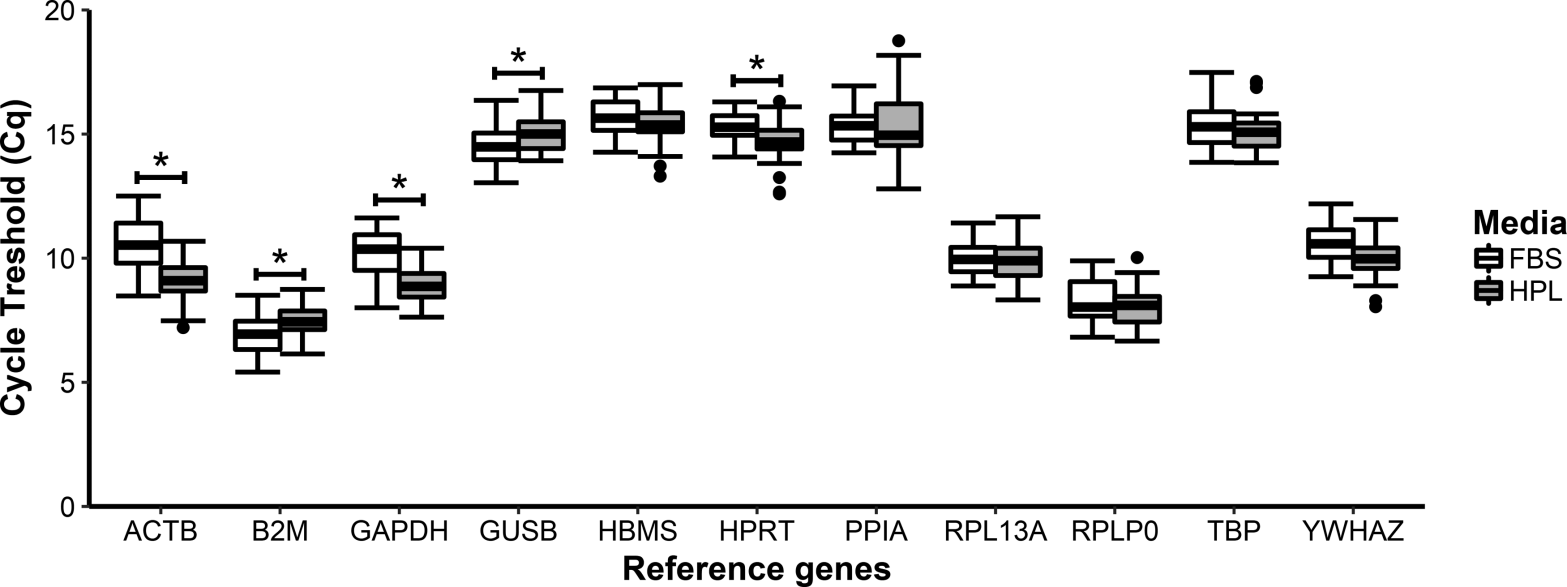
Box and whisker plots of Cq values for the 11 RGs assessed in previously cryopreserved ASCs expanded in FBS and previously cryopreserved ASCs expanded in pHPL. *Boxes* extend from the first to third quartiles with the median shown as the *solid black line* intersecting the *box*; the *whiskers* extend to the minimum and maximum values that lie within 1.5 × the IQR. *Data points* beyond the *whiskers* represent outliers. The sample size is *n* = 36 and represents four biological replicates at nine time points at different differentiation states (day 0, days 1 control and induced, day 7 control and indicated, day 14 control and induced, and day 21 control and induced). *Statistical significance *p* < 0.05.

We examined the effect that expansion medium had on RG expression and stability by grouping all the samples for the frozen FBS and frozen HPL groups. The frozen FBS group had significantly higher Cq values for *ACTB*, *GAPDH*, and *HPRT*, whereas the frozen HPL group had significantly higher Cq values for *B2M* and *GUSB* (Fig. 3).

RG stability was measured for each time point for both control and induced samples for each of the groups (Table 4; Frozen FBS vs. Frozen HPL) using both efficiency methods. When *E* = 2, *ACTB*, *HPRT*, *HBMS*, and *YWHAZ* appeared more regularly in the higher rankings, whereas *GUSB*, *RPLP0*, *RPLP13A* and *PPIA* appeared more regularly in the lower rankings. When the SE was used, *HBMS*, *HPRT*, and *YWHAZ* appeared more regularly in the higher rankings, whereas *RPLP0*, *RPL13A*, and *PPIA* appeared more regularly in the lower ranking. When all the samples were combined and *E* = 2, *HBMS*, *HPRT*, *PPIA*, and *GAPDH* were the most stable and *RPL13A*, *GUSB*, *B2M*, and *RPLP0* were the least stable. When the SE was used, *HPRT*, *YWHAZ*, *HBMS*, and *TBP* were the most stable, whereas *B2M*, *GUSB*, *RPL13A*, and *RPLP0* were the least stable.

## Discussion

ASCs can be cryopreserved for future use with apparently limited alterations to their inherent characteristics.^1,10^ ASCs are being assessed a number of settings in the hope that a cell therapy product will be identified. Independent of their therapeutic efficacy, ASC products need to adhere to GMP standards and the cells should retain their unique cell-specific characteristics.^28^ One of these characteristics is the ability to differentiate into adipocytes, which also provides a valuable tool for understanding the process of adipogenesis in obesity research.^17^

qPCR is used to measure the effects of experimental conditions on gene expression. Guidelines were published to aid researchers in experimental design and quality assurance to produce reproducible data^25,29^: these include the selection of stable RGs. RGs should remain constant in all cells and not change under experimental conditions. Some studies make use of one RG for normalization, which may affect interpretation of results. The use of a geometric mean of multiple RGs for normalization is preferable.^22,30,31^

Many platforms that assess RG stability are available and they use different algorithms to rank the most stable RGs in a given panel.^25,26,32^ Several studies have compared the platforms with one another and have mostly reported that the results are similar between them.^33,34^ The most commonly and widely used platform is geNorm,^25^ which requires the input data to be converted from raw Cq values into relative expression values using the equation *E*^-ΔCq^. A number of studies assume that efficiencies are 100% and set *E =* 2, whereas other studies calculate efficiencies from the standard curves for each of the RGs. The use of different efficiencies has been shown to impact the ranking of RG stability,^33^ especially when the efficiency deviates from its ideal (*E* = 2, 100% efficiency). By using SE, technical variation is taken into consideration and provides a more accurate result. In this study, we observed that the *E* = 2 and SE methods yielded similar results with regard to position changes in the rankings of RGs in the different groups.

The choice of sample grouping or testing subsets differs in the literature.^23,35–37^ Some studies group all of their samples, regardless of the nature of the experiment, and report on the overall RG stability. In a study by Fink *et al.*,^23^ all samples for each passage; hypoxic treatment; and adipogenic, osteogenic, and chondrogenic differentiation were grouped together and the authors reported on the overall stability of the RGs. Other studies have separated samples into experimental groups or subsets. In a study by Li *et al.*,^35^ samples were grouped into separate developmental stages of the different parts of the celery plant; they then combined the development stages and looked at the overall stability in the different parts of the celery plant; and lastly, all samples were combined into one group and overall stability within the celery plant was described.

In our study, we grouped our samples according to the assumptions we make (Table 2) in our adipogenic differentiation assay and how the different experimental conditions affect the adipogenic capacity of ASCs. In our adipogenic differentiation assay,^9,17^ we have investigated changes in gene expression for 21 days. We measured gene expression on different days by normalizing our induced samples to our control samples and then compare the fold changes in gene expression at the different time points. To accurately report on changes in fold expression, we assumed that the RGs are stable and that they do not change significantly between days in either the control or the induced samples. Another assumption we made is that the experimental conditions (medium choice and cryopreservation status) do not affect RG stability. In this study, we have tested these assumptions by measuring the effect that different experimental conditions have on RG stability using both the *E* = 2 and SE methods. More specifically, we examined RG stability (1) at the different time points in the differentiation assay, (2) between control and induced samples, (3) between cryopreservation states, and (4) between different media used for expansion purposes.

Irrespective of the efficiency method or experimental grouping used, we found that all the stability values were <0.5 (Tables 3 and 4), suggesting that all 11 RGs can be considered to be stable, although some ranked better than others. Furthermore, when considering the optimal number of RGs to be used for normalization, all the RG pairwise variation values were <0.15 (Supplementary Tables S3 and S4), suggesting that only two RGs are required for normalization and the addition of more RGs would provide no significant improvement. However, the use of more stable RGs can reduce variations in expression levels and is recommended.^23^

When compared between days and between control and induced samples, we found that only a few RGs had no change in Cq values in the different experimental groups (Supplementary Figs. S5, S6, S7 and Supplementary Table S2). This corresponds with other studies where the effect of time on culture and treatment affects RG stability.^38–40^ When all the time points and control and induced samples were grouped together, the most stable RGs in the fresh FBS group were *TBP*, *YWHAZ*, and *HPRT*; those in the frozen FBS group were *HPRT*, *YWHAZ*, and *HBMS*; and those in the frozen HPL group were *B2M*, *TBP*, and *GUSB*.

Conflicting data have been reported on the effect of cryopreservation on the adipogenic capacity of ASCs. James *et al.*^41^ found that cryopreservation negatively affects adipogenic potential, whereas Yong *et al.*^42^ found that cryopreservation did not affect adipogenic potential in ASCs. Both of these studies made use of *GAPDH* for normalization, but neither indicated how the stability of *GAPDH* was measured. We, therefore, measured the effect of cryopreservation on RG stability by comparing freshly isolated ASCs with previously frozen ASCs, both expanded in FBS. We grouped all the control and induced samples into a fresh FBS or a frozen FBS group. When comparing RGs between the two groups, we found no significant changes in *PPIA* or *RPLP0* Cq values (Fig. 2), even though they consistently ranked as the least stable RGs for all time points in the control and induced samples using both efficiency methods (Table 4). The lack of significance could be explained by the variability seen in the Cq values of *PPIA* in the fresh FBS group and of *RPLP0* in the frozen FBS group, whereas the variability is taken into consideration by the algorithm of the geNorm software and negatively affects their ranking. In contrast, geNorm ranked *HPRT*, *YWHAZ*, *ACTB*, and *TBP* as the most stable RGs, where *HPRT* and *YWHAZ* were ranked identically for both the efficiency methods, and *ACTB* and *TBP* switched positions depending on the efficiency method used.

The use of pHPL for ASC expansion has numerous advantages over FBS.^43–45^ Most studies have shown that pHPL has little or no effect on the adipogenic potential of ASCs when compared with FBS.^46,47^ In our study, freshly isolated ASCs expanded in 10% pHPL detached after a few days in the differentiation assay; as a result we used cryopreserved ASCs expanded in 5% pHPL. The detachment of freshly isolated ASCs expanded in pHPL was also reported by Blande *et al.*^48^ In their study, the authors showed that cryopreserved ASCs expanded in a reduced concentration of pHPL had the ability to differentiate into adipocytes as confirmed by histochemical staining; although this was qualitative evidence, in our study we have used a quantitative approach. Therefore, we investigated whether RG stability differed when the medium was supplemented with either FBS or pHPL in the same cryopreservation state. We grouped control and induced samples of the previously frozen ASCs into either a frozen FBS or a frozen HPL group. When compared, the Cq values of *HBMS*, *PPIA*, *RPL13A*, *RPLP0*, *TBP*, and *YWHAZ* were not significantly different between the frozen FBS and frozen pHPL groups (Fig. 3). When stability was ranked, *HPRT* and *HBMS* were in the top three stable RGs with minor position changes depending on the efficiency method used (Table 4). *YWHAZ* was ranked as one of the most stable genes for the SE method, whereas *PPIA* was ranked as one of the most stable RGs when *E* = 2 was used. *RPLP0* ranked as the least stable when all of the control and induced samples were grouped together, irrespective of the efficiency method used.

When considering the entire study, *PPIA* had the lowest *R*^2^ value, the smallest *E* value, the greatest variability, and regularly appeared toward the bottom of the stability rankings, except when the frozen FBS and frozen HPL groups were compared. These findings are in contrast to those of Fink *et al.* who found that *PPIA* was stable during adipogenesis and Tratwal *et al.*, who found *PPIA* to be stable during cell expansion.^8,23^ In our study, in the fresh FBS groups on the different days in control and induced samples (Table 3), *PPIA* consistently ranked as the least stable RG using both efficiency methods; however, in the frozen FBS and frozen pHPL groups on different days in control and induced samples, *PPIA* showed major position changes in both efficiency methods. Based on these findings, we concluded that *PPIA* should not be considered as a stable RG and consequently should not be used in further studies.

It is well known that primary cells such as ASCs display both inter- and intrapatient variability before and after differentiation.^49^ These variations could affect some of the most important adipocyte functions such as lipid formation, insulin sensitivity, and adipokine function, all of which can affect RG stability. This study did not access these possible differences and should be considered if functional studies are being performed. Furthermore, to prevent experimental bias in our comparisons, the experimental design was identical between all of the experimental conditions.

In this study we did not find any one specific RG that consistently ranked as the most stable in all experimental groups. However, there were RGs that appeared more regularly in the higher rankings. We further found that RG stability differed between days, differentiation status, cryopreservation status, and the expansion medium used. Similar findings were established by Ferguson *et al.* when comparing RG stability in 3T3-L1 adipocytes under different experimental conditions.^50^

## Conclusions

We suggest that the use of RGs for normalization should be selected on the basis of the experiment being performed. For adipogenic differentiation for a 21 day induction period for a single experimental condition (control vs. induced), we suggest using RGs specific to the different groups being assessed: *TBP*, *YWHAZ*, *HPRT*, and *ACTB* for freshly isolated ASCs expanded in FBS; *HPRT*, *YWHAZ*, *HBMS*, and *ACTB* for previously frozen ASCs expanded in FBS; and *B2M*, *GUSB*, *TBP*, and *YWHAZ* for previously frozen ASCs expanded in pHPL. When introducing more than one experimental condition during adipogenic differentiation (e.g., fresh vs thawed ASCs expanded in FBS or pHPL), we propose using *HPRT*, *YWHAZ*, *ACTB*, and *TBP* for comparing fresh versus cryopreserved cells, and *HBMS*, *HPRT*, *YWHAZ*, and *TBP* for the comparison of different media.

## Declarations

### Ethics approval and consent to participate

Informed consent was obtained before the isolation procedure from lipoaspirate, and approval for the study was granted by the University of Pretoria Health Sciences Research Ethics Committee (approval number 421/2013), as well as the Human Research Committee, SANBS, South Africa (2013/17).

## Supplementary Material

Supplemental data

Supplemental data

Supplemental data

Supplemental data

Supplemental data

Supplemental data

Supplemental data

Supplemental data

Supplemental data

Supplemental data

Supplemental data

Supplemental data
