# Supplementary material for: Reference Gene Expression in Adipose-Derived Stromal Cells Undergoing Adipogenic Differentiation
Source: Tissue Eng Part C Methods. 2019 Jun 17;25(6):353–66. doi: 10.1089/ten.tec.2019.0076 (PMC6589494; doi:10.1089/ten.tec.2019.0076)
Supplement: Supplemental data [file Supp_Fig1.pdf]

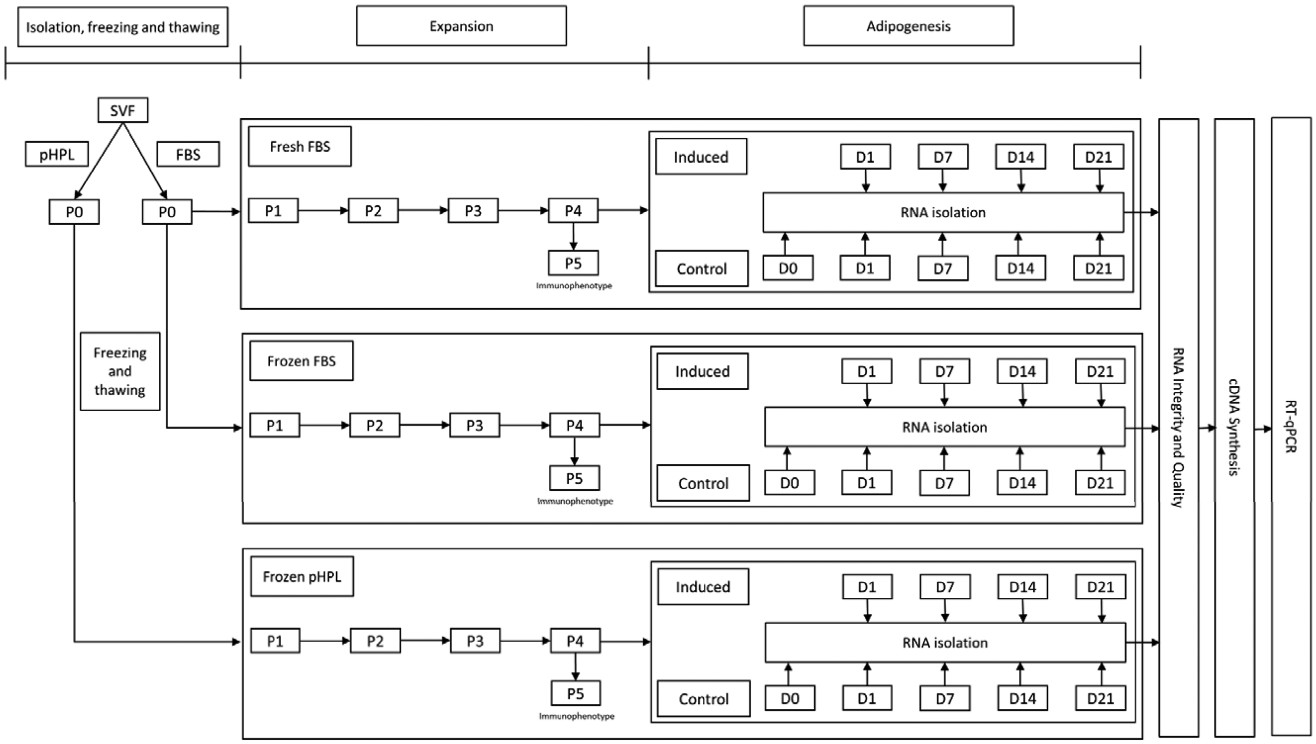

SUPPLEMENTARY FIG. S1. Experimental design and layout. ASCs were freshly isolated from the SVF of four volunteer donors and plated (P0) in either pHPL or FBS supplemented medium. At confluence, cells were dissociated and either plated (P1) for further expansion (fresh FBS) or frozen. The P0 frozen ASCs that had been supplemented with FBS (frozen FBS) or pHPL (frozen pHPL) were thawed and plated (P1). All three experimental groups were maintained/ expanded for four passages whereafter they were plated for further adipogenic induction (P5) and immunophenotypic surface marker expression. When ASCs in the different experimental groups were roughly 80% confluent (day 0; D0), four of the flasks (D1, D7, D14, and D21) were induced using adipogenic medium supplemented with either FBS or pHPL and four were used as controls (D1, D7, D14, and D21). On the respective days (D0, D1, D7, D14, and D21), the cells were dissociated and 1×10^6 cells were used for RNA isolation. RNA integrity and quality were tested and only RNA with an RIN value >8 and absorption ODs >2 was used for cDNA synthesis. The same amount of RNA (100 ng) was used for each condition/time point for cDNA synthesis, and the same starting amount of cDNA (50 ng) was used in the RT-qPCR experiments. ASC, adipose-derived stromal cell; pHPL, pooled human platelet lysate; FBS, fetal bovine serum; RT-qPCR, reverse-transcription quantitative polymerase chain reaction; SVF, stromal vascular fraction.
