# Supplementary material for: Reference Gene Expression in Adipose-Derived Stromal Cells Undergoing Adipogenic Differentiation
Source: Tissue Eng Part C Methods. 2019 Jun 17;25(6):353–66. doi: 10.1089/ten.tec.2019.0076 (PMC6589494; doi:10.1089/ten.tec.2019.0076)
Supplement: Supplemental data [file Supp_Data.pdf]

Supplementary Data

Immunophenotypic Surface Marker Expression

At P5, adipose-derived stromal cell (ASC) surface marker expression or immunophenotype was measured using methods previously described.[S1] The following panel of fluorochrome-conjugated monoclonal antibodies was included: CD73-FITC (eBiosciences, San Diego, CA), CD105-PE (BioLegend, San Diego, CA), CD31 APC (BioLegend), CD44 APC/Cy7 (BioLegend), CD90 BV421 (BD Biosciences, San Jose, CA), and CD45 BV510 (BD Biosciences). At P5, when confluent, ASCs were dissociated and 100 μL of cell suspension was simultaneously stained with 5 μL of each antibody and incubated for 15 min in the dark, wherein 1 mL phosphate buffered saline (PBS) was added to the tubes and centrifuged at 184 *g* for 5 min. The PBS was aspirated and 700 μL PBS was added to the tube and analyzed on a Gallios flow cytometer (Beckman Coulter, Miami, FL). Unstained cells were used to establish the negative limits. The intact ASC population was identified using forward scatter versus side scatter two parameter plots. All subsequent flow cytometric plots were gated on the intact ASC population. The positive expression of the different surface markers was identified using a one parameter log histogram plot, and the coexpression profiles were obtained using tree plots. Flow cytometry data were analyzed postacquisition using Kaluza Post-Acquisition Flow Cytometry Analysis software (Version 1.3). All three experimental groups had >80% positivity for the immunophenotypic profile $CD44^{+}CD45^{-}CD73^{+}CD90^{+}CD105^{+}$, and were negative for the endothelial marker CD 31 (<2%).

Adipocyte Quantification

Detection of intracellular lipid accumulation and adipocyte quantification was performed using flow cytometry, with minor modifications, by costaining induced and noninduced ASCs with Nile red and DAPI, as previously described in our laboratory.[S2] On day 21, the ASCs were dissociated and prepared for staining. Working solutions for both DAPI and Nile red (Sigma-Aldrich Chemie, Steinheim, Germany) were prepared in absolute ethanol (Sigma-Aldrich Chemie). Cell suspensions of both induced and noninduced ASCs were simultaneously stained with 10 μL Nile red (10 μg/mL) and 10 μL DAPI (2 μg/mL) and incubated in the dark for 20 min at RT and, thereafter, analyzed using a Gallios flow cytometer. Differentiated ASCs (adipocytes) were identified as Nile red^{Pos}/$DAPI^{Pos}$ cells to ensure that only intact nucleated cells were assessed. Free-floating lipid droplets ($DAPI^{Neg}$) were excluded from the analysis. All postacquisition analyses were performed using Kaluza Flow Cytometry Analysis Software (Version 1.3).

When compared with noninduced (control) ASCs, induced ASCs in each of the experimental groups underwent adipogenesis (Supplementary Fig. S3) as shown by a significant increase in the positivity of Nile red ($p \leq 0.05$). When comparing induced ASCs between experimental groups, the frozen pooled human platelet lysate (pHPL) group had significantly less adipogenesis ($p = 0.006$) when compared with the frozen fetal bovine serum (FBS) group. Induced ASCs in the fresh FBS group and frozen FBS group did not show significant differences in Nile red positivity, neither did induced ASCs of the fresh FBS group and the frozen pHPL group.

Fluorescence Microscopy

Intracellular lipid droplets were visualized by costaining with Nile red and VDC violet (Thermo Fisher Scientific/Life technologies; Waltham, MA) and captured using fluorescence imaging (Supplementary Fig. S4). On day 21, ASCs were prepared for staining. A working solution of 50 ng/μL Nile red was prepared in absolute ethanol and 10 μL was gently added to ASCs, followed by the addition of 2 μL VDC violet. The ASCs were then incubated at 37°C in 5% CO_2 for 20 min. Thereafter, images were captured with a 20× magnification objective lens using an AxioVert A1 inverted fluorescence microscope equipped with an AxioCam Cm1 camera (Carl Zeiss, Cottigen, Germany). Each image represents an overlay image converted from three single channel images, where the first two channels captured Nile red staining (visualization of lipid droplet formation; yellow green fluorescence, filter set 9; and deep red fluorescence, filter set 00) and the last channel captured VDC violet staining (visualization of nuclei; DAPI fluorescence, filter set 49). Images were captured using AxioVision software (Version 4.8.2; Carl Zeiss) and analyzed postacquisition using Image J imaging software (Version 1.49). Images were enhanced for contrast and brightness and were not manipulated.

Effect of Kinetics and Adipogenic Differentiation on Reference Gene Expression

When comparing the effect of differentiation between control and induced samples in each group, significant differences were found in the fresh FBS group (Supplementary Fig. S5), on day 7 for *B2M* ($p = 0.03$) and *GUSB* ($p = 0.03$); and on day 21 for *ACTB* ($p = 0.03$), *B2M* ($p = 0.03$), *GUSB* ($p = 0.03$), and *YWHAZ* ($p = 0.03$). For the frozen FBS group (Supplementary Fig. S6), significant differences between the control and the induced samples were observed on day 7 for *B2M* ($p = 0.03$), and on day 21 for *ACTB* ($p = 0.03$) and *B2M* ($p = 0.03$). For the frozen *HPL* group (Supplementary Fig. S7), significant differences were found on day 1 for *GAPDH* ($p = 0.03$) and *RPLP0* ($p = 0.03$), on day 7 for *HPRT* ($p = 0.03$) and *RPL13A* ($p = 0.03$), and on day 21 for *YWHAZ* ($p = 0.03$).

When considering kinetics in the differentiation assay, significant changes were observed in the Cq values of the fresh FBS control samples (Supplementary Fig. S5) from day 0 to day 1 for *ACTB* ($p = 0.002$), *GAPDH* ($p = 0.005$), *HBMS* ($p = 0.04$), *HPRT* ($p = 0.01$), *RPLP0* ($p = 0.04$), *TBP* ($p = 0.003$), and *YWHAZ* ($p = 0.01$); from day 0 to day 21 for *B2M* ($p = 0.006$), *GUSB* ($p = 0.03$), and *RPL13A* ($p = 0.009$); and from day 1 to day 14 for *GAPDH* ($p = 0.04$), *HBMS* ($p = 0.02$), *HPRT* ($p = 0.01$), *RPLP0* ($p = 0.005$), *TBP* ($p = 0.04$), and *YWHAZ* ($p = 0.02$). In contrast, no change

was seen in induced samples at the different time points for the fresh FBS group. In the frozen FBS group (Supplementary Fig. S6), significant changes were observed in induced samples between day 0 and day 14 for *B2M* ($p=0.03$), *GAPDH* ($p=0.04$), *HBMS* ($p=0.04$), and *YWHAZ* ($p=0.02$); on day 21 for *B2M* ($p=0.02$); between day 1 and day 14 ($p=0.006$) and day 1 and day 21 ($p=0.01$) for *ACTB*; and between day 1 and 7 for *PPIA* ($p=0.04$). Furthermore, a difference in Cq values in the control samples was observed between day 0 and day 7 for *HPRT* ($p=0.02$) and between day 1 and day 7 for *ACTB* ($p=0.004$), *HPRT* ($p=0.02$), and *PPIA* ($p=0.04$). In the frozen HPL group (Supplementary Fig. S7), significant changes in the Cq values were observed in induced samples between days 0 and 21 for *ACTB* ($p=0.003$), *B2M* ($p=0.04$), *GAPDH* ($p=0.03$), *HBMS* ($p=0.02$), *HPRT* ($p=0.02$), and *YWHAZ* ($p=0.02$); and between day 1 and day 21 for *GAPDH* ($p=0.02$).

Supplementary References

S1. Dessels, C., Durandt, C., and Pepper, M.S. Comparison of human platelet lysate alternatives using expired and freshly isolated platelet concentrates for adipose-derived stromal cell expansion. Platelets **30**, 356, 2019.

S2. Durandt, C., van Vollenstee, F.A., Dessels, C., *et al.* Novel flow cytometric approach for the detection of adipocyte sub-populations during adipogenesis. J Lipid Res **57,** 729, 2016.
