# Supplementary material for: Reference Gene Expression in Adipose-Derived Stromal Cells Undergoing Adipogenic Differentiation
Source: Tissue Eng Part C Methods. 2019 Jun 17;25(6):353–66. doi: 10.1089/ten.tec.2019.0076 (PMC6589494; doi:10.1089/ten.tec.2019.0076)
Supplement: Supplemental data [file Supp_Table1.pdf]

Supplementary Table S1. Percentage Viability of the Noninduced (Control) and Induced ASCs Undergoing Adipogenesis For 21 Days Before RNA Isolation

	Fresh FBS		*Frozen FBS*		*Frozen pHPL*	
	Control	*Induced*	*Control*	*Induced*	*Control*	*Induced*
Day 0	97.58 ± (0.53)	—	95.85 ± (0.38)	—	98.84 ± (0.91)	—
Day 1	96.58 ± (0.76)	96.61 ± (0.68)	95.71 ± (0.37)	95.96 ± (0.09)	98.91 ± (1.17)	98.24 ± (2.17)
Day 7	91.66 ± (2.71)	88.91 ± (4.23)	92.98 ± (0.79)	95.00 ± (0.45)	96.00 ± (3.50)	98.27 ± (1.16)
Day 14	77.51 ± (7.67)	75.05 ± (8.09)	72.23 ± (2.85)	89.28 ± (1.72)	82.67 ± (14.19)	96.91 ± (2.65)
Day 21	56.01 ± (11.25)	64.32 ± (8.15)	57.77 ± (3.23)	77.29 ± (4.17)	90.13 ± (9.77)	93.68 ± (10.64)

Viability was measured using 7-AAD staining techniques.
ASC, adipose-derived stromal cell; pHPL, pooled human platelet lysate; FBS, fetal bovine serum.
