# Supplementary material for: Reference Gene Expression in Adipose-Derived Stromal Cells Undergoing Adipogenic Differentiation
Source: Tissue Eng Part C Methods. 2019 Jun 17;25(6):353–66. doi: 10.1089/ten.tec.2019.0076 (PMC6589494; doi:10.1089/ten.tec.2019.0076)
Supplement: Supplemental data [file Supp_Fig3.pdf]

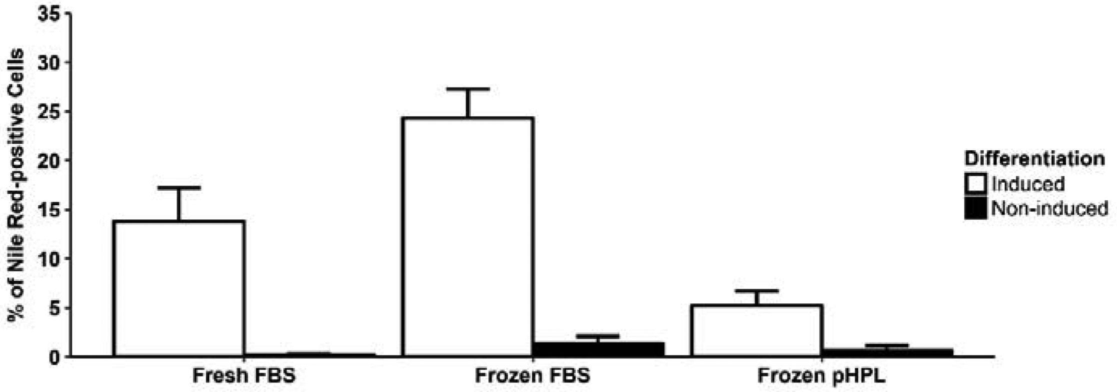

SUPPLEMENTARY FIG. S3. Percentage of ASCs undergoing adipogenesis in freshly isolated ASCs expanded in FBS (fresh FBS), previously cryopreserved ASCs expanded in FBS (frozen FBS), and previously cryopreserved ASCs expanded in pHPL (frozen HPL). Bars represent the mean ± standard error and the sample size is $n = 4$.
