# Supplementary material for: Reference Gene Expression in Adipose-Derived Stromal Cells Undergoing Adipogenic Differentiation
Source: Tissue Eng Part C Methods. 2019 Jun 17;25(6):353–66. doi: 10.1089/ten.tec.2019.0076 (PMC6589494; doi:10.1089/ten.tec.2019.0076)
Supplement: Supplemental data [file Supp_Fig4.pdf]

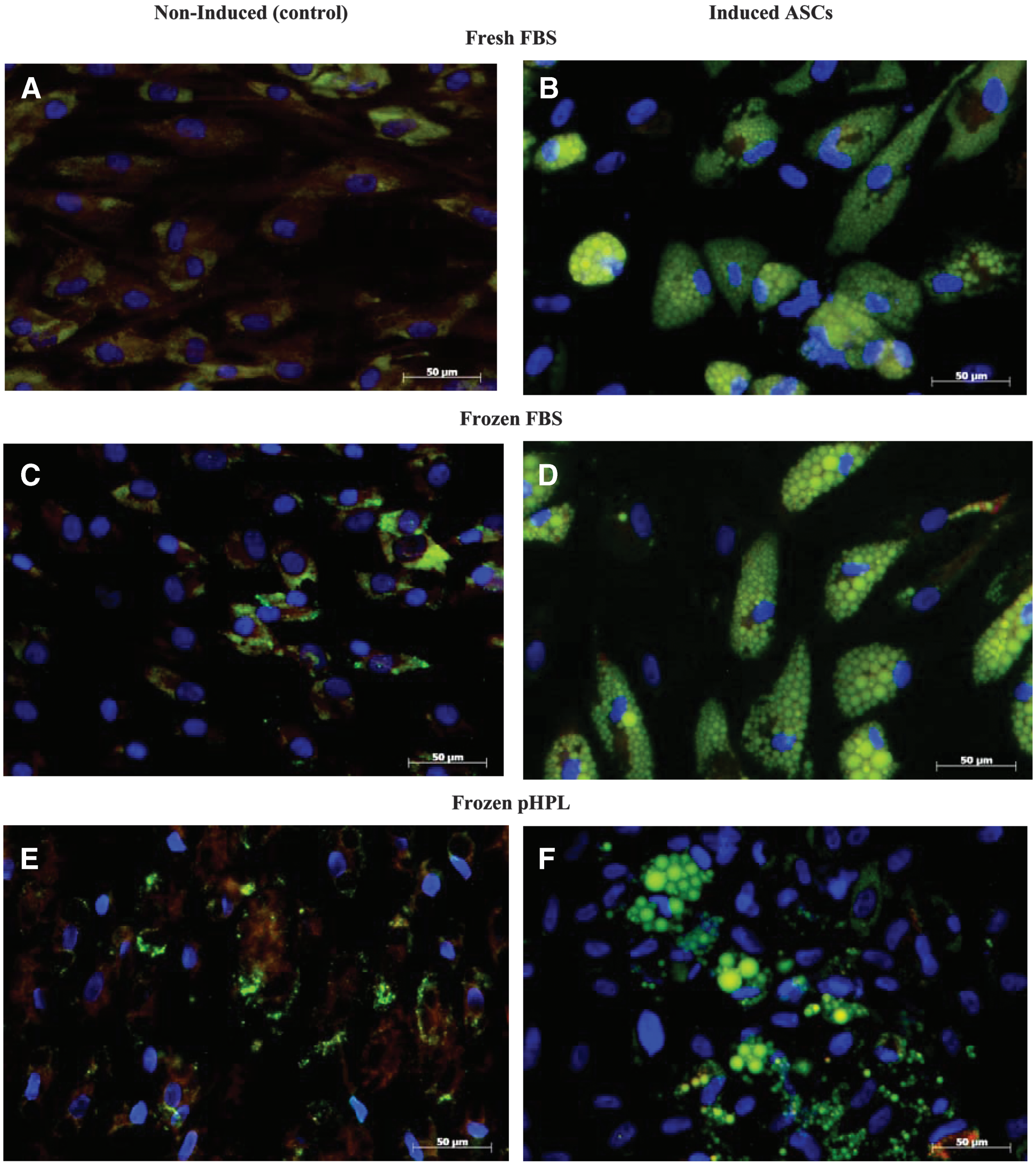

SUPPLEMENTARY FIG. S4. Fluorescence microscopy images of day 21 noninduced and induced **(A, B)** freshly isolated ASCs expanded in FBS (fresh FBS), **(C, D)** previously cryopreserved ASCs expanded in FBS (frozen FBS), and **(E, F)** previously cryopreserved ASCs expanded in pHPL (frozen HPL). Nuclei were stained with VDC violet and the lipid droplets were stained with Nile red. Images were captured at 20× magnification.
