# Supplementary material for: Reference Gene Expression in Adipose-Derived Stromal Cells Undergoing Adipogenic Differentiation
Source: Tissue Eng Part C Methods. 2019 Jun 17;25(6):353–66. doi: 10.1089/ten.tec.2019.0076 (PMC6589494; doi:10.1089/ten.tec.2019.0076)
Supplement: Supplemental data [file Supp_Fig6.pdf]

Previously frozen ASCs expanded in FBS

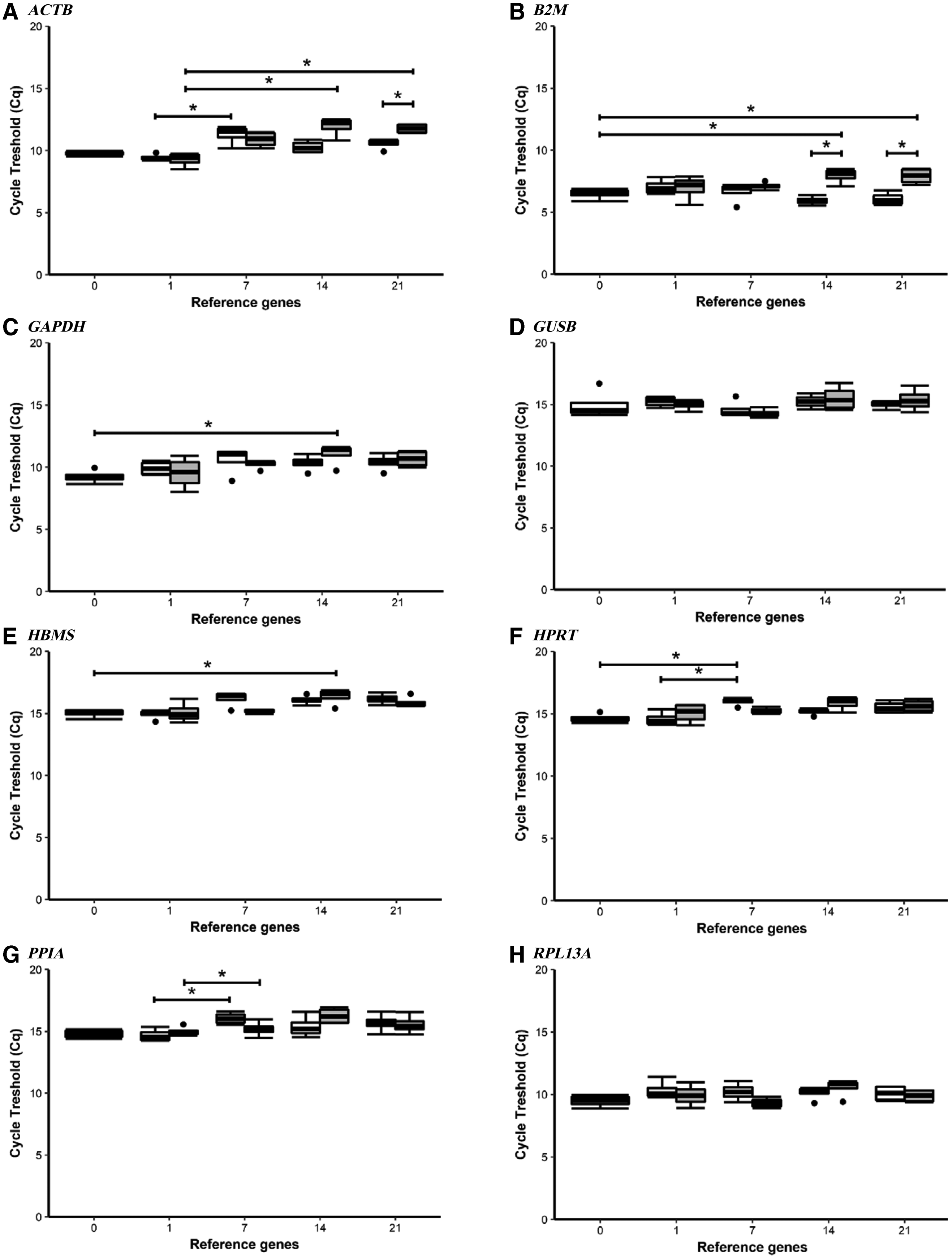

SUPPLEMENTARY FIG. S6. Box and whisker plots of the Cq values for the 11 RGs for previously frozen ASCs expanded in FBS. The 11 RGs are **(A)** *ACTB*, **(B)** *B2M*, **(C)** *GAPDH*, **(D)** *GUSB*, **(E)** *HBMS*, **(F)** *HPRT*, **(G)** *PPIA*, **(H)** *RPL13A*, **(I)** *RPLP0*, **(J)** *TBP*, and **(K)** *YWHAZ*. *Boxes* extend from the first to third quartiles with the median shown as the *solid black line* intersecting the *box*, the *whiskers* extend to the minimum and maximum values that lie within 1.5 × the IQR. *Data points* beyond the *whiskers* represent outliers. The sample size is $n = 4$ and statistical significance is represented by $p < 0.05$ (*). *White boxes* represent the noninduced ASCs and the *gray boxes* represent the induced ASCs.

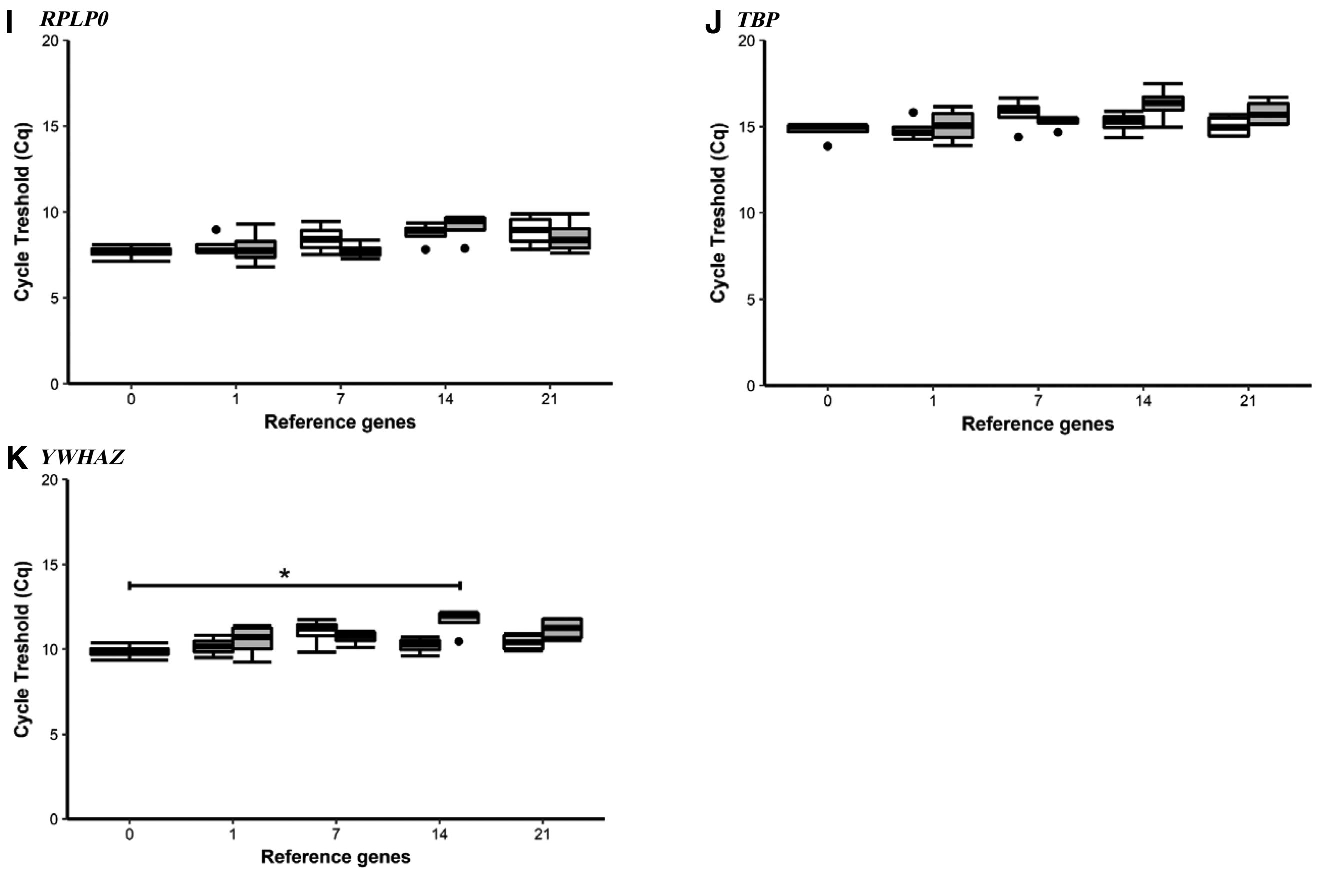

SUPPLEMENTARY FIG. S6. (*Continued*).
