# Supplementary material for: Reference Gene Expression in Adipose-Derived Stromal Cells Undergoing Adipogenic Differentiation
Source: Tissue Eng Part C Methods. 2019 Jun 17;25(6):353–66. doi: 10.1089/ten.tec.2019.0076 (PMC6589494; doi:10.1089/ten.tec.2019.0076)
Supplement: Supplemental data [file Supp_Fig7.pdf]

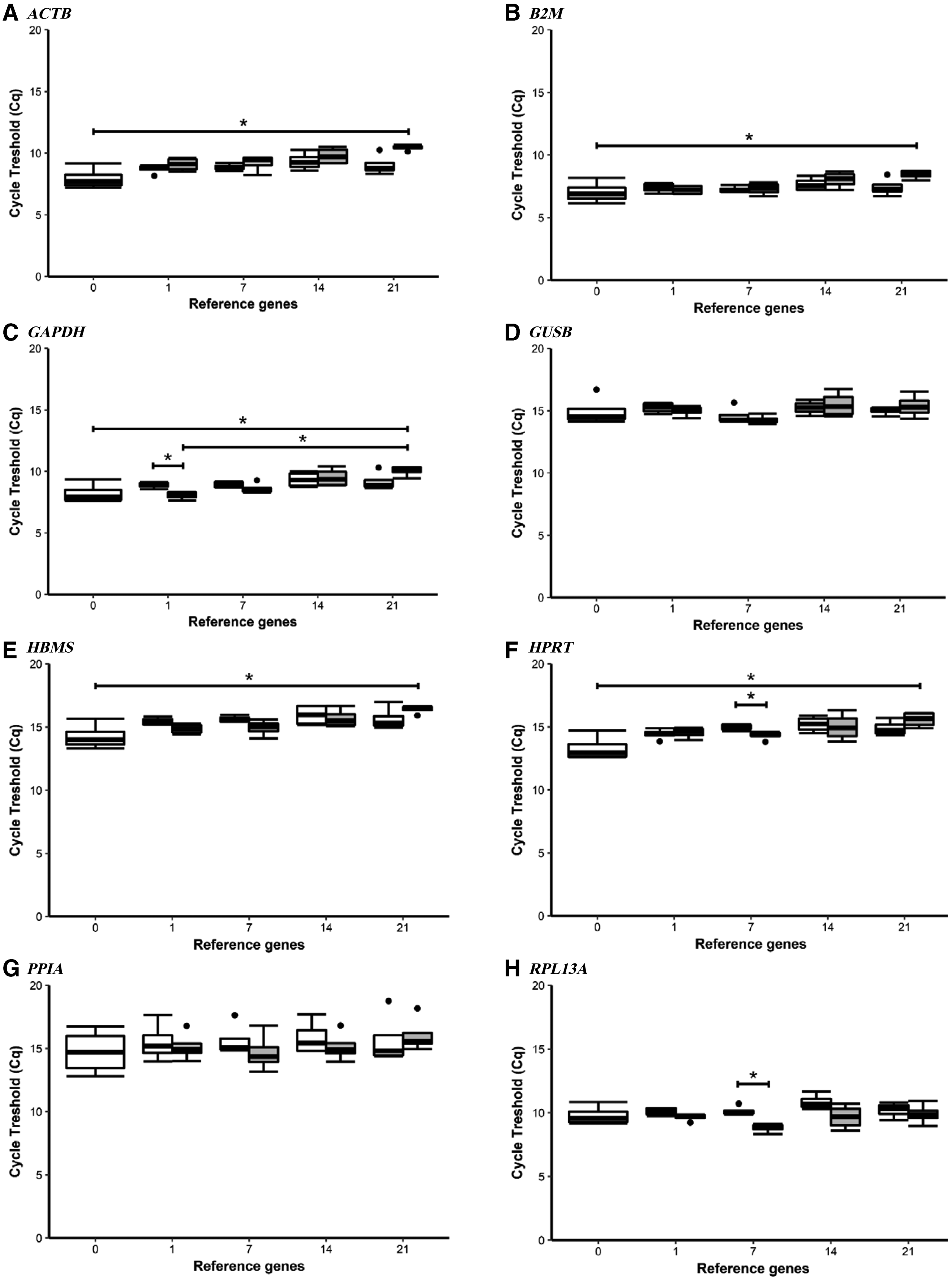

SUPPLEMENTARY FIG. S7. Box and whisker plots of the Cq values for the 11 RGs for previously frozen ASCs expanded in pHPL. The 11 RGs are **(A)** *ACTB*, **(B)** *B2M*, **(C)** *GAPDH*, **(D)** *GUSB*, **(E)** *HBMS*, **(F)** *HPRT*, **(G)** *PPIA*, **(H)** *RPL13A*, **(I)** *RPLP0*, **(J)** *TBP*, and **(K)** *YWHAZ*. *Boxes* extend from the first to third quartiles with the median shown as the *solid black line* intersecting the *box*, the *whiskers* extend to the minimum and maximum values that lie within 1.5 × the IQR. *Data points* beyond the *whiskers* represent outliers. The sample size is $n = 4$ and statistical significance is represented by $p < 0.05$ (*). *White boxes* represent the noninduced ASCs and the *gray boxes* represent the induced ASCs.

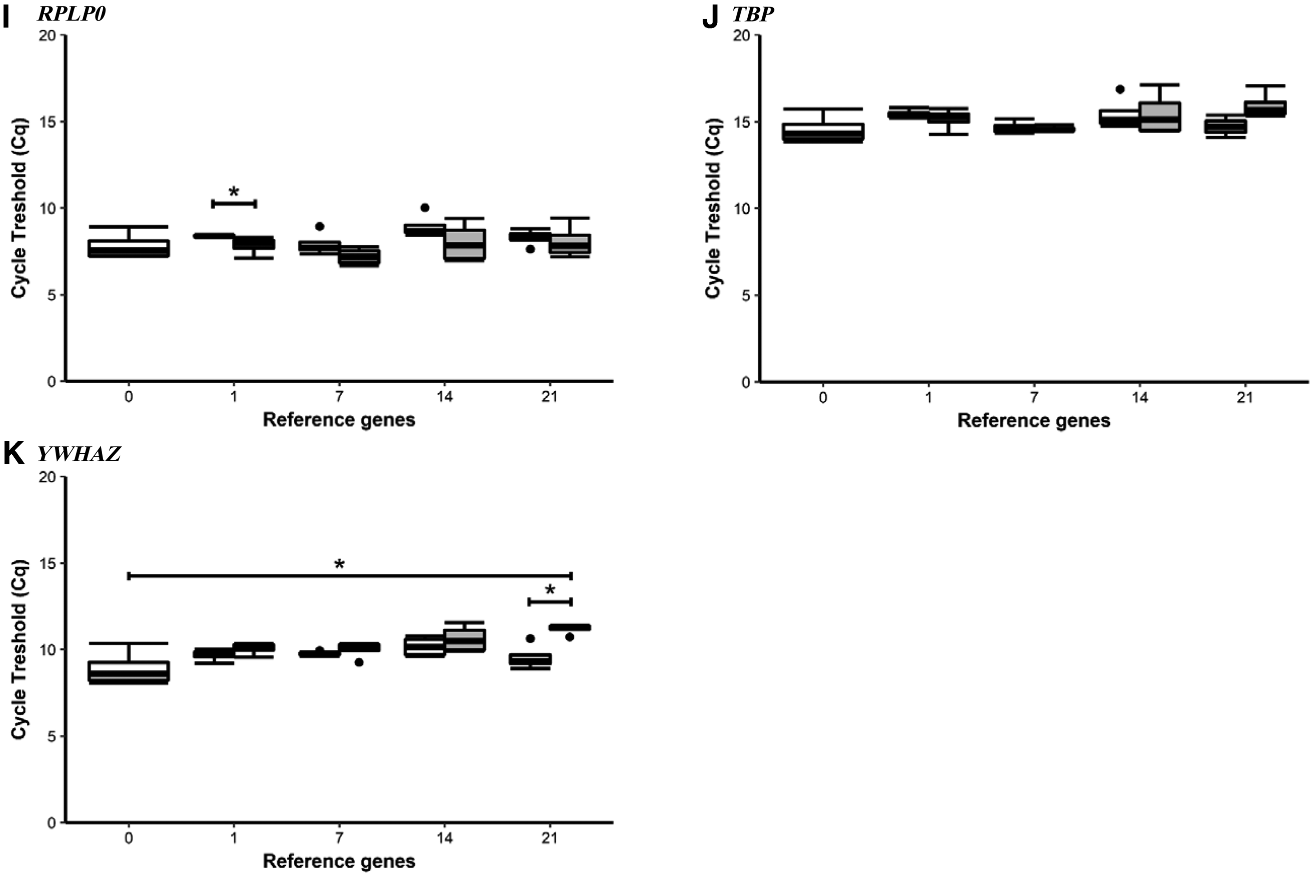

SUPPLEMENTARY FIG. S7. (*Continued*).
