# Supplementary material for: Reference Gene Expression in Adipose-Derived Stromal Cells Undergoing Adipogenic Differentiation
Source: Tissue Eng Part C Methods. 2019 Jun 17;25(6):353–66. doi: 10.1089/ten.tec.2019.0076 (PMC6589494; doi:10.1089/ten.tec.2019.0076)
Supplement: Supplemental data [file Supp_Table2.docx]

**Table S2: Descriptive statistics of Cq values for each reference gene (RG) for each condition (media type, cryopreservation status, differentiation status and day of induction).**

| **Media** | **Cryo.** | **Day** | **Diff.** | **ACTB** | | | | | | **B2M** | | | | | |
| --- | --- | --- | --- | --- | --- | --- | --- | --- | --- | --- | --- | --- | --- | --- | --- |
|  |  |  |  | **Mean** | **Min** | **Max** | **SD** | **Var** | **SE** | **Mean** | **Min** | **Max** | **SD** | **Var** | **SE** |
| **FBS** | **Fresh** | **0** | **Control** | 8.95 | 8.24 | 9.27 | 0.48 | 0.23 | 0.24 | 5.97 | 5.60 | 6.44 | 0.35 | 0.13 | 0.18 |
|  |  | **1** | **Control** | 7.24 | 6.91 | 7.70 | 0.33 | 0.11 | 0.17 | 4.97 | 4.62 | 5.47 | 0.36 | 0.13 | 0.18 |
|  |  |  | **Induced** | 8.41 | 7.94 | 9.32 | 0.79 | 0.63 | 0.46 | 5.28 | 4.62 | 6.10 | 0.75 | 0.57 | 0.43 |
|  |  | **7** | **Control** | 8.70 | 8.15 | 9.71 | 0.71 | 0.51 | 0.36 | 4.77 | 4.45 | 5.33 | 0.38 | 0.15 | 0.19 |
|  |  |  | **Induced** | 10.03 | 8.16 | 12.32 | 1.80 | 3.24 | 0.90 | 6.80 | 5.59 | 8.48 | 1.37 | 1.89 | 0.69 |
|  |  | **14** | **Control** | 9.04 | 8.38 | 10.37 | 0.92 | 0.85 | 0.46 | 4.96 | 4.52 | 5.93 | 0.66 | 0.43 | 0.33 |
|  |  |  | **Induced** | 10.06 | 8.87 | 11.02 | 1.12 | 1.25 | 0.56 | 6.79 | 5.77 | 8.05 | 1.14 | 1.30 | 0.57 |
|  |  | **21** | **Control** | 8.50 | 8.22 | 8.93 | 0.32 | 0.11 | 0.16 | 4.42 | 4.19 | 4.70 | 0.24 | 0.06 | 0.12 |
|  |  |  | **Induced** | 9.50 | 8.94 | 9.95 | 0.42 | 0.18 | 0.21 | 6.09 | 5.56 | 6.42 | 0.41 | 0.17 | 0.20 |
| **FBS** | **Frozen** | **0** | **Control** | 9.77 | 9.53 | 9.98 | 0.18 | 0.03 | 0.09 | 6.49 | 5.88 | 6.89 | 0.43 | 0.19 | 0.22 |
|  |  | **1** | **Control** | 9.43 | 9.19 | 9.82 | 0.27 | 0.07 | 0.14 | 7.03 | 6.48 | 7.83 | 0.60 | 0.36 | 0.30 |
|  |  |  | **Induced** | 9.27 | 8.48 | 9.75 | 0.57 | 0.33 | 0.29 | 6.97 | 5.59 | 7.88 | 0.99 | 0.99 | 0.50 |
|  |  | **7** | **Control** | 11.28 | 10.19 | 11.90 | 0.76 | 0.58 | 0.38 | 6.62 | 5.41 | 7.19 | 0.82 | 0.66 | 0.41 |
|  |  |  | **Induced** | 10.89 | 10.20 | 11.47 | 0.62 | 0.38 | 0.31 | 7.11 | 6.77 | 7.50 | 0.30 | 0.09 | 0.15 |
|  |  | **14** | **Control** | 10.28 | 9.86 | 10.89 | 0.50 | 0.25 | 0.25 | 5.94 | 5.55 | 6.37 | 0.34 | 0.11 | 0.17 |
|  |  |  | **Induced** | 11.93 | 10.81 | 12.50 | 0.78 | 0.61 | 0.39 | 7.94 | 7.09 | 8.48 | 0.61 | 0.37 | 0.31 |
|  |  | **21** | **Control** | 10.56 | 9.92 | 10.89 | 0.44 | 0.19 | 0.22 | 6.06 | 5.57 | 6.75 | 0.53 | 0.28 | 0.26 |
|  |  |  | **Induced** | 11.76 | 11.40 | 12.09 | 0.37 | 0.14 | 0.19 | 7.91 | 7.19 | 8.51 | 0.65 | 0.43 | 0.33 |
| **HPL** | **Frozen** | **0** | **Control** | 7.95 | 7.20 | 9.17 | 0.87 | 0.76 | 0.43 | 7.03 | 6.14 | 8.19 | 0.87 | 0.77 | 0.44 |
|  |  | **1** | **Control** | 8.72 | 8.16 | 9.02 | 0.38 | 0.15 | 0.19 | 7.40 | 6.93 | 7.77 | 0.38 | 0.15 | 0.19 |
|  |  |  | **Induced** | 9.09 | 8.50 | 9.61 | 0.53 | 0.28 | 0.27 | 7.22 | 6.89 | 7.54 | 0.36 | 0.13 | 0.18 |
|  |  | **7** | **Control** | 8.87 | 8.56 | 9.22 | 0.28 | 0.08 | 0.14 | 7.24 | 7.05 | 7.60 | 0.25 | 0.06 | 0.13 |
|  |  |  | **Induced** | 9.19 | 8.23 | 9.64 | 0.66 | 0.44 | 0.33 | 7.30 | 6.70 | 7.81 | 0.49 | 0.24 | 0.25 |
|  |  | **14** | **Control** | 9.34 | 8.60 | 10.27 | 0.72 | 0.52 | 0.36 | 7.66 | 7.21 | 8.35 | 0.54 | 0.29 | 0.27 |
|  |  |  | **Induced** | 9.78 | 9.19 | 10.53 | 0.69 | 0.47 | 0.34 | 8.03 | 7.20 | 8.68 | 0.66 | 0.43 | 0.33 |
|  |  | **21** | **Control** | 9.04 | 8.34 | 10.26 | 0.84 | 0.71 | 0.42 | 7.43 | 6.71 | 8.44 | 0.73 | 0.53 | 0.36 |
|  |  |  | **Induced** | 10.45 | 10.13 | 10.68 | 0.23 | 0.05 | 0.12 | 8.47 | 7.99 | 8.74 | 0.35 | 0.12 | 0.18 |

**Table S2. Continued.**

| **Media** | **Cryo.** | **Day** | **Diff.** | **GAPDH** | | | | | | **GUSB** | | | | | |
| --- | --- | --- | --- | --- | --- | --- | --- | --- | --- | --- | --- | --- | --- | --- | --- |
|  |  |  |  | **Mean** | **Min** | **Max** | **SD** | **Var** | **SE** | **Mean** | **Min** | **Max** | **SD** | **Var** | **SE** |
| **FBS** | **Fresh** | **0** | **Control** | 8.17 | 7.83 | 8.58 | 0.34 | 0.12 | 0.17 | 14.06 | 13.70 | 14.33 | 0.27 | 0.07 | 0.14 |
|  |  | **1** | **Control** | 7.08 | 6.84 | 7.35 | 0.26 | 0.07 | 0.13 | 12.87 | 12.36 | 13.58 | 0.56 | 0.32 | 0.28 |
|  |  |  | **Induced** | 7.41 | 6.71 | 8.01 | 0.66 | 0.43 | 0.38 | 13.13 | 12.39 | 13.91 | 0.76 | 0.58 | 0.44 |
|  |  | **7** | **Control** | 7.67 | 7.44 | 7.89 | 0.18 | 0.03 | 0.09 | 12.84 | 12.42 | 13.07 | 0.30 | 0.09 | 0.15 |
|  |  |  | **Induced** | 9.29 | 7.55 | 11.30 | 1.66 | 2.74 | 0.83 | 14.81 | 13.53 | 16.84 | 1.46 | 2.14 | 0.73 |
|  |  | **14** | **Control** | 8.15 | 7.62 | 9.20 | 0.71 | 0.50 | 0.35 | 13.25 | 12.58 | 14.33 | 0.75 | 0.56 | 0.38 |
|  |  |  | **Induced** | 9.02 | 7.97 | 9.85 | 0.96 | 0.93 | 0.48 | 14.91 | 13.66 | 16.18 | 1.23 | 1.52 | 0.62 |
|  |  | **21** | **Control** | 7.75 | 7.51 | 8.03 | 0.21 | 0.05 | 0.11 | 12.62 | 12.12 | 12.97 | 0.36 | 0.13 | 0.18 |
|  |  |  | **Induced** | 8.30 | 8.01 | 8.52 | 0.23 | 0.05 | 0.12 | 14.11 | 13.78 | 14.32 | 0.24 | 0.06 | 0.12 |
| **FBS** | **Frozen** | **0** | **Control** | 9.23 | 8.64 | 9.95 | 0.54 | 0.29 | 0.27 | 14.29 | 13.29 | 14.77 | 0.68 | 0.46 | 0.34 |
|  |  | **1** | **Control** | 9.92 | 9.39 | 10.53 | 0.57 | 0.32 | 0.28 | 14.27 | 13.52 | 15.38 | 0.79 | 0.62 | 0.39 |
|  |  |  | **Induced** | 9.53 | 8.00 | 10.91 | 1.30 | 1.68 | 0.65 | 14.03 | 13.04 | 15.04 | 0.85 | 0.73 | 0.43 |
|  |  | **7** | **Control** | 10.56 | 8.90 | 11.24 | 1.12 | 1.25 | 0.56 | 14.21 | 13.32 | 15.10 | 0.73 | 0.54 | 0.37 |
|  |  |  | **Induced** | 10.24 | 9.70 | 10.49 | 0.36 | 0.13 | 0.18 | 14.25 | 13.85 | 14.74 | 0.37 | 0.14 | 0.18 |
|  |  | **14** | **Control** | 10.35 | 9.50 | 11.06 | 0.64 | 0.41 | 0.32 | 14.23 | 13.35 | 14.66 | 0.59 | 0.35 | 0.30 |
|  |  |  | **Induced** | 11.04 | 9.72 | 11.62 | 0.89 | 0.79 | 0.44 | 15.72 | 14.64 | 16.36 | 0.75 | 0.56 | 0.37 |
|  |  | **21** | **Control** | 10.41 | 9.51 | 11.14 | 0.67 | 0.45 | 0.34 | 14.27 | 13.49 | 15.05 | 0.77 | 0.59 | 0.38 |
|  |  |  | **Induced** | 10.67 | 9.97 | 11.30 | 0.66 | 0.44 | 0.33 | 15.37 | 14.83 | 15.85 | 0.42 | 0.18 | 0.21 |
| **HPL** | **Frozen** | **0** | **Control** | 8.22 | 7.63 | 9.37 | 0.80 | 0.64 | 0.40 | 14.97 | 14.14 | 16.70 | 1.17 | 1.37 | 0.59 |
|  |  | **1** | **Control** | 8.89 | 8.55 | 9.12 | 0.25 | 0.06 | 0.12 | 15.24 | 14.73 | 15.63 | 0.43 | 0.18 | 0.21 |
|  |  |  | **Induced** | 8.06 | 7.64 | 8.33 | 0.32 | 0.10 | 0.16 | 14.99 | 14.40 | 15.37 | 0.42 | 0.17 | 0.21 |
|  |  | **7** | **Control** | 8.94 | 8.70 | 9.19 | 0.25 | 0.06 | 0.12 | 14.59 | 14.14 | 15.65 | 0.72 | 0.51 | 0.36 |
|  |  |  | **Induced** | 8.60 | 8.30 | 9.29 | 0.46 | 0.21 | 0.23 | 14.28 | 13.93 | 14.78 | 0.36 | 0.13 | 0.18 |
|  |  | **14** | **Control** | 9.34 | 8.74 | 10.02 | 0.64 | 0.41 | 0.32 | 15.25 | 14.59 | 15.88 | 0.56 | 0.31 | 0.28 |
|  |  |  | **Induced** | 9.50 | 8.87 | 10.40 | 0.74 | 0.55 | 0.37 | 15.50 | 14.57 | 16.75 | 1.01 | 1.03 | 0.51 |
|  |  | **21** | **Control** | 9.18 | 8.64 | 10.31 | 0.77 | 0.59 | 0.38 | 15.02 | 14.56 | 15.26 | 0.32 | 0.10 | 0.16 |
|  |  |  | **Induced** | 10.06 | 9.44 | 10.34 | 0.42 | 0.18 | 0.21 | 15.37 | 14.37 | 16.55 | 0.92 | 0.85 | 0.46 |

**Table S2. Continued.**

| **Media** | **Cryo.** | **Day** | **Diff.** | **HBMS** | | | | | | **HPRT** | | | | | |
| --- | --- | --- | --- | --- | --- | --- | --- | --- | --- | --- | --- | --- | --- | --- | --- |
|  |  |  |  | **Mean** | **Min** | **Max** | **SD** | **Var** | **SE** | **Mean** | **Min** | **Max** | **SD** | **Var** | **SE** |
| **FBS** | **Fresh** | **0** | **Control** | 14.47 | 13.63 | 15.29 | 0.73 | 0.53 | 0.36 | 13.74 | 13.30 | 14.06 | 0.33 | 0.11 | 0.16 |
|  |  | **1** | **Control** | 12.90 | 12.53 | 13.29 | 0.40 | 0.16 | 0.20 | 12.26 | 11.92 | 12.84 | 0.43 | 0.18 | 0.21 |
|  |  |  | **Induced** | 13.20 | 12.69 | 13.99 | 0.69 | 0.48 | 0.40 | 13.24 | 12.63 | 13.82 | 0.60 | 0.35 | 0.34 |
|  |  | **7** | **Control** | 13.80 | 13.26 | 14.09 | 0.38 | 0.15 | 0.19 | 13.22 | 12.89 | 13.81 | 0.41 | 0.17 | 0.20 |
|  |  |  | **Induced** | 14.94 | 13.03 | 17.10 | 1.71 | 2.91 | 0.85 | 14.64 | 12.71 | 16.58 | 1.62 | 2.62 | 0.81 |
|  |  | **14** | **Control** | 14.55 | 13.90 | 15.38 | 0.62 | 0.39 | 0.31 | 14.01 | 13.31 | 15.19 | 0.82 | 0.67 | 0.41 |
|  |  |  | **Induced** | 15.12 | 13.78 | 16.59 | 1.53 | 2.34 | 0.76 | 14.65 | 13.43 | 16.35 | 1.42 | 2.01 | 0.71 |
|  |  | **21** | **Control** | 14.24 | 13.87 | 14.67 | 0.33 | 0.11 | 0.17 | 13.34 | 12.99 | 13.72 | 0.32 | 0.10 | 0.16 |
|  |  |  | **Induced** | 14.48 | 14.03 | 14.73 | 0.31 | 0.10 | 0.16 | 13.73 | 13.54 | 14.11 | 0.26 | 0.07 | 0.13 |
| **FBS** | **Frozen** | **0** | **Control** | 15.01 | 14.53 | 15.26 | 0.33 | 0.11 | 0.17 | 14.62 | 14.26 | 15.14 | 0.37 | 0.14 | 0.19 |
|  |  | **1** | **Control** | 14.93 | 14.34 | 15.24 | 0.40 | 0.16 | 0.20 | 14.57 | 14.12 | 15.37 | 0.56 | 0.32 | 0.28 |
|  |  |  | **Induced** | 15.07 | 14.27 | 16.19 | 0.82 | 0.68 | 0.41 | 15.04 | 14.08 | 15.69 | 0.79 | 0.63 | 0.40 |
|  |  | **7** | **Control** | 16.18 | 15.23 | 16.60 | 0.64 | 0.40 | 0.32 | 15.98 | 15.51 | 16.27 | 0.33 | 0.11 | 0.16 |
|  |  |  | **Induced** | 15.17 | 14.94 | 15.32 | 0.17 | 0.03 | 0.08 | 15.25 | 15.01 | 15.58 | 0.25 | 0.06 | 0.12 |
|  |  | **14** | **Control** | 16.10 | 15.64 | 16.57 | 0.38 | 0.14 | 0.19 | 15.18 | 14.79 | 15.42 | 0.27 | 0.07 | 0.14 |
|  |  |  | **Induced** | 16.36 | 15.41 | 16.86 | 0.66 | 0.43 | 0.33 | 15.87 | 15.11 | 16.30 | 0.55 | 0.30 | 0.27 |
|  |  | **21** | **Control** | 16.18 | 15.69 | 16.68 | 0.41 | 0.17 | 0.21 | 15.51 | 15.10 | 16.09 | 0.48 | 0.23 | 0.24 |
|  |  |  | **Induced** | 15.89 | 15.64 | 16.59 | 0.46 | 0.21 | 0.23 | 15.63 | 15.12 | 16.20 | 0.51 | 0.26 | 0.26 |
| **HPL** | **Frozen** | **0** | **Control** | 14.24 | 13.31 | 15.67 | 1.03 | 1.07 | 0.52 | 13.30 | 12.59 | 14.71 | 0.98 | 0.96 | 0.49 |
|  |  | **1** | **Control** | 15.44 | 15.19 | 15.84 | 0.30 | 0.09 | 0.15 | 14.43 | 13.85 | 14.88 | 0.43 | 0.18 | 0.21 |
|  |  |  | **Induced** | 14.85 | 14.43 | 15.26 | 0.40 | 0.16 | 0.20 | 14.55 | 13.96 | 14.93 | 0.43 | 0.19 | 0.22 |
|  |  | **7** | **Control** | 15.63 | 15.43 | 15.97 | 0.25 | 0.06 | 0.13 | 14.97 | 14.65 | 15.21 | 0.25 | 0.06 | 0.13 |
|  |  |  | **Induced** | 14.93 | 14.10 | 15.59 | 0.63 | 0.40 | 0.32 | 14.34 | 13.82 | 14.63 | 0.36 | 0.13 | 0.18 |
|  |  | **14** | **Control** | 15.95 | 15.16 | 16.66 | 0.83 | 0.69 | 0.42 | 15.21 | 14.49 | 15.89 | 0.64 | 0.40 | 0.32 |
|  |  |  | **Induced** | 15.68 | 15.05 | 16.67 | 0.74 | 0.54 | 0.37 | 15.00 | 13.83 | 16.32 | 1.11 | 1.22 | 0.55 |
|  |  | **21** | **Control** | 15.65 | 14.95 | 16.99 | 0.92 | 0.85 | 0.46 | 14.89 | 14.34 | 15.71 | 0.61 | 0.37 | 0.31 |
|  |  |  | **Induced** | 16.40 | 15.92 | 16.64 | 0.33 | 0.11 | 0.16 | 15.57 | 14.89 | 16.10 | 0.60 | 0.37 | 0.30 |

**Table S2. Continued.**

| **Media** | **Cryo.** | **Day** | **Diff.** | **PPIA** | | | | | | **RPL13A** | | | | | |
| --- | --- | --- | --- | --- | --- | --- | --- | --- | --- | --- | --- | --- | --- | --- | --- |
|  |  |  |  | **Mean** | **Min** | **Max** | **SD** | **Var** | **SE** | **Mean** | **Min** | **Max** | **SD** | **Var** | **SE** |
| **FBS** | **Fresh** | **0** | **Control** | 14.04 | 12.91 | 15.89 | 1.32 | 1.76 | 0.66 | 9.50 | 9.15 | 10.04 | 0.38 | 0.15 | 0.19 |
|  |  | **1** | **Control** | 12.38 | 11.47 | 13.83 | 1.01 | 1.02 | 0.51 | 8.55 | 8.29 | 8.82 | 0.22 | 0.05 | 0.11 |
|  |  |  | **Induced** | 13.26 | 12.14 | 14.15 | 1.03 | 1.05 | 0.59 | 8.47 | 7.75 | 9.13 | 0.69 | 0.48 | 0.40 |
|  |  | **7** | **Control** | 13.21 | 12.31 | 14.51 | 0.94 | 0.89 | 0.47 | 8.61 | 8.12 | 9.12 | 0.41 | 0.17 | 0.20 |
|  |  |  | **Induced** | 14.46 | 12.55 | 16.17 | 1.67 | 2.80 | 0.84 | 9.20 | 7.84 | 10.88 | 1.29 | 1.66 | 0.64 |
|  |  | **14** | **Control** | 14.20 | 13.22 | 16.18 | 1.37 | 1.87 | 0.68 | 9.05 | 8.57 | 9.70 | 0.47 | 0.22 | 0.24 |
|  |  |  | **Induced** | 14.88 | 12.23 | 18.63 | 2.75 | 7.58 | 1.38 | 9.06 | 7.92 | 10.52 | 1.28 | 1.65 | 0.64 |
|  |  | **21** | **Control** | 13.64 | 12.27 | 15.64 | 1.43 | 2.06 | 0.72 | 8.35 | 8.16 | 8.57 | 0.22 | 0.05 | 0.11 |
|  |  |  | **Induced** | 13.74 | 12.42 | 15.89 | 1.51 | 2.29 | 0.76 | 8.29 | 8.00 | 8.62 | 0.28 | 0.08 | 0.14 |
| **FBS** | **Frozen** | **0** | **Control** | 14.79 | 14.41 | 15.15 | 0.34 | 0.11 | 0.17 | 9.48 | 8.88 | 9.97 | 0.48 | 0.23 | 0.24 |
|  |  | **1** | **Control** | 14.68 | 14.24 | 15.36 | 0.51 | 0.26 | 0.25 | 10.33 | 9.75 | 11.42 | 0.75 | 0.56 | 0.38 |
|  |  |  | **Induced** | 14.98 | 14.66 | 15.56 | 0.40 | 0.16 | 0.20 | 9.93 | 8.93 | 10.99 | 0.88 | 0.77 | 0.44 |
|  |  | **7** | **Control** | 16.05 | 15.54 | 16.61 | 0.49 | 0.24 | 0.24 | 10.22 | 9.40 | 11.08 | 0.71 | 0.50 | 0.35 |
|  |  |  | **Induced** | 15.19 | 14.47 | 15.99 | 0.62 | 0.39 | 0.31 | 9.33 | 8.92 | 9.82 | 0.40 | 0.16 | 0.20 |
|  |  | **14** | **Control** | 15.38 | 14.53 | 16.58 | 0.88 | 0.77 | 0.44 | 10.14 | 9.31 | 10.53 | 0.56 | 0.31 | 0.28 |
|  |  |  | **Induced** | 16.25 | 15.68 | 16.94 | 0.66 | 0.43 | 0.33 | 10.55 | 9.41 | 11.06 | 0.77 | 0.59 | 0.38 |
|  |  | **21** | **Control** | 15.68 | 14.75 | 16.59 | 0.75 | 0.56 | 0.38 | 10.08 | 9.48 | 10.64 | 0.63 | 0.40 | 0.32 |
|  |  |  | **Induced** | 15.54 | 14.74 | 16.56 | 0.76 | 0.58 | 0.38 | 9.89 | 9.39 | 10.33 | 0.51 | 0.26 | 0.26 |
| **HPL** | **Frozen** | **0** | **Control** | 14.73 | 12.79 | 16.75 | 1.82 | 3.33 | 0.91 | 9.79 | 9.16 | 10.84 | 0.76 | 0.57 | 0.38 |
|  |  | **1** | **Control** | 15.50 | 13.98 | 17.63 | 1.55 | 2.41 | 0.78 | 10.10 | 9.74 | 10.37 | 0.32 | 0.10 | 0.16 |
|  |  |  | **Induced** | 15.15 | 14.00 | 16.79 | 1.17 | 1.37 | 0.59 | 9.64 | 9.23 | 9.82 | 0.28 | 0.08 | 0.14 |
|  |  | **7** | **Control** | 15.66 | 14.86 | 17.63 | 1.32 | 1.74 | 0.66 | 10.14 | 9.88 | 10.72 | 0.39 | 0.15 | 0.20 |
|  |  |  | **Induced** | 14.68 | 13.17 | 16.80 | 1.53 | 2.34 | 0.77 | 8.81 | 8.32 | 9.14 | 0.35 | 0.13 | 0.18 |
|  |  | **14** | **Control** | 15.84 | 14.81 | 17.70 | 1.37 | 1.87 | 0.68 | 10.85 | 10.31 | 11.67 | 0.60 | 0.36 | 0.30 |
|  |  |  | **Induced** | 15.14 | 13.95 | 16.82 | 1.20 | 1.45 | 0.60 | 9.67 | 8.61 | 10.71 | 0.95 | 0.91 | 0.48 |
|  |  | **21** | **Control** | 15.69 | 14.37 | 18.76 | 2.07 | 4.30 | 1.04 | 10.21 | 9.43 | 10.82 | 0.60 | 0.36 | 0.30 |
|  |  |  | **Induced** | 16.07 | 14.95 | 18.17 | 1.43 | 2.06 | 0.72 | 9.90 | 8.96 | 10.93 | 0.80 | 0.65 | 0.40 |

**Table S2. Continued.**

| **Media** | **Cryo.** | **Day** | **Diff.** | **RPLP0** | | | | | | **TBP** | | | | | |
| --- | --- | --- | --- | --- | --- | --- | --- | --- | --- | --- | --- | --- | --- | --- | --- |
|  |  |  |  | **Mean** | **Min** | **Max** | **SD** | **Var** | **SE** | **Mean** | **Min** | **Max** | **SD** | **Var** | **SE** |
| **FBS** | **Fresh** | **0** | **Control** | 8.24 | 7.79 | 8.92 | 0.49 | 0.24 | 0.24 | 14.54 | 14.14 | 14.75 | 0.27 | 0.07 | 0.14 |
|  |  | **1** | **Control** | 7.16 | 6.64 | 7.36 | 0.35 | 0.12 | 0.17 | 12.98 | 12.73 | 13.29 | 0.25 | 0.06 | 0.13 |
|  |  |  | **Induced** | 7.17 | 6.55 | 7.80 | 0.62 | 0.39 | 0.36 | 13.97 | 13.44 | 14.49 | 0.53 | 0.28 | 0.30 |
|  |  | **7** | **Control** | 7.66 | 7.01 | 7.93 | 0.44 | 0.19 | 0.22 | 13.75 | 13.27 | 14.38 | 0.46 | 0.21 | 0.23 |
|  |  |  | **Induced** | 8.38 | 6.92 | 9.96 | 1.36 | 1.85 | 0.68 | 15.48 | 13.95 | 17.58 | 1.59 | 2.52 | 0.79 |
|  |  | **14** | **Control** | 8.51 | 8.00 | 9.00 | 0.42 | 0.18 | 0.21 | 14.26 | 13.84 | 15.18 | 0.63 | 0.39 | 0.31 |
|  |  |  | **Induced** | 8.65 | 7.46 | 9.66 | 1.03 | 1.05 | 0.51 | 15.31 | 14.16 | 16.46 | 1.27 | 1.61 | 0.64 |
|  |  | **21** | **Control** | 7.76 | 7.56 | 8.05 | 0.24 | 0.06 | 0.12 | 13.64 | 13.14 | 14.06 | 0.40 | 0.16 | 0.20 |
|  |  |  | **Induced** | 8.01 | 7.64 | 8.55 | 0.38 | 0.15 | 0.19 | 14.10 | 13.97 | 14.23 | 0.14 | 0.02 | 0.07 |
| **FBS** | **Frozen** | **0** | **Control** | 7.66 | 7.14 | 8.10 | 0.40 | 0.16 | 0.20 | 14.75 | 13.86 | 15.12 | 0.59 | 0.35 | 0.30 |
|  |  | **1** | **Control** | 8.03 | 7.66 | 8.97 | 0.63 | 0.40 | 0.32 | 14.85 | 14.27 | 15.83 | 0.68 | 0.46 | 0.34 |
|  |  |  | **Induced** | 7.90 | 6.82 | 9.30 | 1.04 | 1.08 | 0.52 | 15.05 | 13.89 | 16.16 | 1.03 | 1.06 | 0.52 |
|  |  | **7** | **Control** | 8.45 | 7.52 | 9.46 | 0.84 | 0.70 | 0.42 | 15.74 | 14.39 | 16.66 | 0.96 | 0.92 | 0.48 |
|  |  |  | **Induced** | 7.73 | 7.27 | 8.36 | 0.46 | 0.21 | 0.23 | 15.26 | 14.67 | 15.52 | 0.40 | 0.16 | 0.20 |
|  |  | **14** | **Control** | 8.76 | 7.82 | 9.37 | 0.66 | 0.44 | 0.33 | 15.21 | 14.35 | 15.89 | 0.65 | 0.42 | 0.33 |
|  |  |  | **Induced** | 9.14 | 7.88 | 9.72 | 0.86 | 0.74 | 0.43 | 16.30 | 14.97 | 17.48 | 1.03 | 1.06 | 0.52 |
|  |  | **21** | **Control** | 8.90 | 7.82 | 9.89 | 0.95 | 0.89 | 0.47 | 15.02 | 14.45 | 15.71 | 0.65 | 0.43 | 0.33 |
|  |  |  | **Induced** | 8.56 | 7.62 | 9.89 | 1.00 | 1.00 | 0.50 | 15.80 | 15.10 | 16.69 | 0.78 | 0.60 | 0.39 |
| **HPL** | **Frozen** | **0** | **Control** | 7.80 | 7.21 | 8.92 | 0.79 | 0.63 | 0.40 | 14.55 | 13.84 | 15.73 | 0.84 | 0.71 | 0.42 |
|  |  | **1** | **Control** | 8.38 | 8.32 | 8.44 | 0.06 | 0.00 | 0.03 | 15.45 | 15.21 | 15.81 | 0.26 | 0.07 | 0.13 |
|  |  |  | **Induced** | 7.84 | 7.12 | 8.30 | 0.51 | 0.26 | 0.26 | 15.15 | 14.28 | 15.77 | 0.63 | 0.40 | 0.31 |
|  |  | **7** | **Control** | 7.92 | 7.34 | 8.94 | 0.70 | 0.49 | 0.35 | 14.66 | 14.33 | 15.18 | 0.37 | 0.14 | 0.18 |
|  |  |  | **Induced** | 7.19 | 6.66 | 7.74 | 0.50 | 0.25 | 0.25 | 14.59 | 14.43 | 14.82 | 0.17 | 0.03 | 0.08 |
|  |  | **14** | **Control** | 8.95 | 8.43 | 10.03 | 0.72 | 0.52 | 0.36 | 15.46 | 14.75 | 16.88 | 0.97 | 0.93 | 0.48 |
|  |  |  | **Induced** | 8.00 | 6.96 | 9.39 | 1.16 | 1.34 | 0.58 | 15.46 | 14.47 | 17.12 | 1.25 | 1.56 | 0.63 |
|  |  | **21** | **Control** | 8.30 | 7.62 | 8.79 | 0.49 | 0.24 | 0.25 | 14.72 | 14.09 | 15.39 | 0.56 | 0.31 | 0.28 |
|  |  |  | **Induced** | 8.06 | 7.17 | 9.42 | 0.99 | 0.98 | 0.49 | 15.93 | 15.34 | 17.05 | 0.77 | 0.60 | 0.39 |

**Table S2. Continued.**

| **Media** | **Cryo.** | **Day** | **Diff.** | **YWHAZ** | | | | | |
| --- | --- | --- | --- | --- | --- | --- | --- | --- | --- |
|  |  |  |  | **Mean** | **Min** | **Max** | **SD** | **Var** | **SE** |
| **FBS** | **Fresh** | **0** | **Control** | 8.82 | 8.31 | 9.47 | 0.49 | 0.24 | 0.24 |
|  |  | **1** | **Control** | 7.35 | 7.19 | 7.56 | 0.17 | 0.03 | 0.09 |
|  |  |  | **Induced** | 8.68 | 8.04 | 9.34 | 0.65 | 0.42 | 0.38 |
|  |  | **7** | **Control** | 8.26 | 7.90 | 8.94 | 0.47 | 0.22 | 0.24 |
|  |  |  | **Induced** | 9.90 | 8.26 | 11.75 | 1.53 | 2.36 | 0.77 |
|  |  | **14** | **Control** | 8.84 | 8.29 | 9.61 | 0.57 | 0.33 | 0.29 |
|  |  |  | **Induced** | 9.95 | 8.87 | 11.12 | 1.15 | 1.33 | 0.58 |
|  |  | **21** | **Control** | 8.37 | 7.96 | 8.66 | 0.31 | 0.09 | 0.15 |
|  |  |  | **Induced** | 9.11 | 8.89 | 9.34 | 0.19 | 0.03 | 0.09 |
| **FBS** | **Frozen** | **0** | **Control** | 9.86 | 9.35 | 10.36 | 0.42 | 0.17 | 0.21 |
|  |  | **1** | **Control** | 10.17 | 9.51 | 10.84 | 0.57 | 0.32 | 0.28 |
|  |  |  | **Induced** | 10.53 | 9.25 | 11.40 | 0.98 | 0.96 | 0.49 |
|  |  | **7** | **Control** | 11.02 | 9.83 | 11.74 | 0.83 | 0.69 | 0.41 |
|  |  |  | **Induced** | 10.69 | 10.10 | 11.06 | 0.43 | 0.19 | 0.22 |
|  |  | **14** | **Control** | 10.22 | 9.62 | 10.72 | 0.48 | 0.23 | 0.24 |
|  |  |  | **Induced** | 11.67 | 10.47 | 12.19 | 0.81 | 0.65 | 0.40 |
|  |  | **21** | **Control** | 10.42 | 9.89 | 10.93 | 0.51 | 0.26 | 0.26 |
|  |  |  | **Induced** | 11.21 | 10.51 | 11.81 | 0.67 | 0.45 | 0.34 |
| **HPL** | **Frozen** | **0** | **Control** | 8.90 | 8.05 | 10.37 | 1.04 | 1.09 | 0.52 |
|  |  | **1** | **Control** | 9.68 | 9.20 | 10.03 | 0.35 | 0.12 | 0.18 |
|  |  |  | **Induced** | 10.07 | 9.56 | 10.37 | 0.36 | 0.13 | 0.18 |
|  |  | **7** | **Control** | 9.77 | 9.62 | 9.95 | 0.14 | 0.02 | 0.07 |
|  |  |  | **Induced** | 10.02 | 9.25 | 10.35 | 0.51 | 0.26 | 0.26 |
|  |  | **14** | **Control** | 10.17 | 9.60 | 10.79 | 0.59 | 0.34 | 0.29 |
|  |  |  | **Induced** | 10.62 | 9.92 | 11.56 | 0.80 | 0.63 | 0.40 |
|  |  | **21** | **Control** | 9.55 | 8.90 | 10.64 | 0.76 | 0.57 | 0.38 |
|  |  |  | **Induced** | 11.20 | 10.74 | 11.43 | 0.31 | 0.10 | 0.16 |

*Cryo = Cryopreservation status, diff. = differentiation status, Min = Minimum Cq value, Max = Maximum Cq value, SD = standard deviation of Cq values, Var = Variation of Cq values and SE = Standard error of Cq values.
