# Supplementary material for: Reference Gene Expression in Adipose-Derived Stromal Cells Undergoing Adipogenic Differentiation
Source: Tissue Eng Part C Methods. 2019 Jun 17;25(6):353–66. doi: 10.1089/ten.tec.2019.0076 (PMC6589494; doi:10.1089/ten.tec.2019.0076)
Supplement: Supplemental data [file Supp_Table3.pdf]

Supplementary Table S3. Optimal Number of Reference Gene Determinations Using Pairwise Variation as Performed in the geNorm Package for Freshly Isolated ASCs Expanded in FBS (Fresh FBS), Previously Cryopreserved ASCs Expanded in FBS (Frozen FBS), and Previously Cryopreserved ASCs Expanded in pHPL (Frozen HPL) for Each Time Point and Each Differentiation State

			Fresh FBS		*Frozen FBS*		*pHPL*	
Day	*Differentiation*	*PV*	*100% efficiency*	*Specific efficiency*	*100% efficiency*	*Specific efficiency*	*100% efficiency*	*Specific efficiency*
0	Control	2/3	0.009	0.006	0.013	0.013	0.013	0.014
		3/4	0.008	0.009	0.01	0.008	0.009	0.013
		4/5	0.007	0.007	0.01	0.008	0.009	0.013
		5/6	0.007	0.007	0.009	0.008	0.008	0.01
		6/7	0.007	0.008	0.007	0.01	0.01	0.01
		7/8	0.007	0.006	0.01	0.008	0.012	0.012
		8/9	0.008	0.008	0.011	0.008	0.01	0.011
		9/10	0.01	0.007	0.009	0.007	0.01	0.013
		10/11	0.009	0.014	0.008	0.01	0.01	0.012
1	Control	2/3	0.023	0.027	0.007	0.007	0.006	0.004
		3/4	0.018	0.019	0.005	0.005	0.005	0.004
		4/5	0.015	0.015	0.006	0.005	0.005	0.006
		5/6	0.019	0.02	0.007	0.005	0.005	0.004
		6/7	0.015	0.015	0.006	0.005	0.004	0.003
		7/8	0.013	0.014	0.006	0.005	0.004	0.003
		8/9	0.016	0.015	0.007	0.007	0.004	0.004
		9/10	0.016	0.017	0.009	0.007	0.005	0.006
		10/11	0.033	0.03	0.008	0.009	0.005	0.009
1	Induced	2/3	0.014	0.004	0.008	0.006	0.008	0.006
		3/4	0.01	0.021	0.012	0.008	0.006	0.005
		4/5	0.016	0.018	0.009	0.011	0.007	0.006
		5/6	0.019	0.014	0.011	0.009	0.007	0.005
		6/7	0.014	0.015	0.008	0.008	0.008	0.005
		7/8	0.012	0.014	0.008	0.007	0.006	0.006
		8/9	0.013	0.014	0.007	0.007	0.006	0.006
		9/10	0.013	0.014	0.011	0.01	0.01	0.01
		10/11	0.014	0.014	0.016	0.016	0.012	0.014
7	Control	2/3	0.016	0.026	0.006	0.01	0.004	0.003
		3/4	0.017	0.02	0.008	0.008	0.007	0.007
		4/5	0.013	0.017	0.008	0.007	0.005	0.005
		5/6	0.017	0.016	0.01	0.007	0.005	0.004
		6/7	0.013	0.013	0.009	0.006	0.006	0.004
		7/8	0.013	0.013	0.009	0.007	0.005	0.004
		8/9	0.014	0.012	0.009	0.006	0.005	0.004
		9/10	0.013	0.014	0.008	0.007	0.006	0.005
		10/11	0.026	0.027	0.01	0.009	0.013	0.01
7	Induced	2/3	0.008	0.01	0.005	0.004	0.009	0.009
		3/4	0.011	0.013	0.005	0.006	0.008	0.009
		4/5	0.009	0.011	0.004	0.004	0.011	0.009
		5/6	0.011	0.014	0.004	0.005	0.011	0.009
		6/7	0.009	0.014	0.005	0.005	0.011	0.011
		7/8	0.011	0.013	0.006	0.005	0.009	0.01
		8/9	0.013	0.012	0.005	0.008	0.009	0.016
		9/10	0.011	0.01	0.007	0.007	0.014	0.016
		10/11	0.013	0.015	0.011	0.009	0.022	0.024
14	Control	2/3	0.009	0.01	0.005	0.006	0.005	0.005
		3/4	0.01	0.01	0.005	0.005	0.004	0.004
		4/5	0.008	0.009	0.005	0.005	0.005	0.005
		5/6	0.008	0.008	0.008	0.007	0.003	0.004
		6/7	0.006	0.007	0.008	0.006	0.003	0.003
		7/8	0.008	0.007	0.007	0.006	0.004	0.004
		8/9	0.011	0.01	0.008	0.006	0.006	0.006
		9/10	0.012	0.014	0.007	0.008	0.005	0.005
		10/11	0.012	0.017	0.009	0.012	0.004	0.005

(*continued*)

Supplementary Table S3. (Continued)

			Fresh FBS		*Frozen FBS*		*pHPL*	
Day	*Differentiation*	*PV*	*100% efficiency*	*Specific efficiency*	*100% efficiency*	*Specific efficiency*	*100% efficiency*	*Specific efficiency*
14	Induced	2/3	0.011	0.008	0.003	0.003	0.003	0.004
		3/4	0.007	0.009	0.003	0.003	0.005	0.004
		4/5	0.006	0.008	0.003	0.003	0.003	0.004
		5/6	0.007	0.007	0.002	0.003	0.006	0.006
		6/7	0.009	0.008	0.003	0.004	0.008	0.008
		7/8	0.009	0.007	0.004	0.004	0.008	0.007
		8/9	0.009	0.008	0.005	0.006	0.01	0.013
		9/10	0.015	0.02	0.007	0.006	0.013	0.012
		10/11	0.021	0.022	0.009	0.009	0.021	0.018
21	Control	2/3	0.008	0.007	0.005	0.005	0.005	0.007
		3/4	0.007	0.007	0.005	0.005	0.007	0.007
		4/5	0.012	0.012	0.005	0.005	0.006	0.005
		5/6	0.008	0.01	0.008	0.007	0.006	0.005
		6/7	0.007	0.009	0.008	0.006	0.006	0.005
		7/8	0.01	0.013	0.007	0.005	0.005	0.005
		8/9	0.01	0.013	0.008	0.006	0.005	0.006
		9/10	0.012	0.013	0.007	0.007	0.005	0.006
		10/11	0.027	0.032	0.009	0.01	0.008	0.008
21	Induced	2/3	0.009	0.009	0.003	0.004	0.002	0.003
		3/4	0.007	0.007	0.003	0.003	0.001	0.001
		4/5	0.008	0.007	0.003	0.004	0.004	0.003
		5/6	0.007	0.007	0.002	0.004	0.003	0.003
		6/7	0.008	0.008	0.003	0.004	0.003	0.003
		7/8	0.011	0.011	0.004	0.004	0.005	0.005
		8/9	0.011	0.011	0.005	0.005	0.005	0.006
		9/10	0.012	0.013	0.007	0.005	0.011	0.01
		10/11	0.017	0.022	0.009	0.011	0.017	0.015
	All	2/3	0.025	0.019	0.014	0.01	0.021	0.017
		3/4	0.02	0.021	0.01	0.008	0.017	0.017
		4/5	0.019	0.023	0.009	0.008	0.013	0.012
		5/6	0.018	0.02	0.008	0.008	0.011	0.013
		6/7	0.019	0.019	0.01	0.009	0.01	0.01
		7/8	0.02	0.019	0.01	0.008	0.009	0.011
		8/9	0.022	0.02	0.009	0.009	0.01	0.011
		9/10	0.02	0.02	0.008	0.009	0.013	0.012
		10/11	0.018	0.025	0.012	0.011	0.016	0.014

PV, pairwise variation value.
