# Supplementary material for: Reference Gene Expression in Adipose-Derived Stromal Cells Undergoing Adipogenic Differentiation
Source: Tissue Eng Part C Methods. 2019 Jun 17;25(6):353–66. doi: 10.1089/ten.tec.2019.0076 (PMC6589494; doi:10.1089/ten.tec.2019.0076)
Supplement: Supplemental data [file Supp_Table4.pdf]

Supplementary Table S4. Optimal Number of Reference Gene Determination Using Pairwise Variation as Performed in the geNorm Package for the Fresh FBS and Frozen FBS Groups, and the Frozen FBS and Frozen HPL Groups for Each Time Point and Each Differentiation State

			Frozen pHPL vs. frozen FBS		*Frozen FBS vs. fresh FBS*	
Day	*Differentiation*	*Rank*	*100% efficiency*	*Specific efficiency*	*100% efficiency*	*Specific efficiency*
0	Control	2/3	0.016	0.017	0.016	0.015
		3/4	0.024	0.015	0.012	0.011
		4/5	0.020	0.013	0.011	0.010
		5/6	0.019	0.022	0.009	0.009
		6/7	0.014	0.016	0.013	0.011
		7/8	0.012	0.012	0.010	0.010
		8/9	0.012	0.014	0.009	0.008
		9/10	0.014	0.013	0.008	0.013
		10/11	0.018	0.018	0.014	0.013
1	Control	2/3	0.012	0.011	0.021	0.024
		3/4	0.009	0.009	0.019	0.019
		4/5	0.010	0.008	0.018	0.018
		5/6	0.009	0.009	0.016	0.016
		6/7	0.008	0.008	0.018	0.022
		7/8	0.009	0.008	0.017	0.017
		8/9	0.009	0.008	0.016	0.016
		9/10	0.009	0.009	0.019	0.020
		10/11	0.008	0.009	0.022	0.020
1	Induced	2/3	0.011	0.010	0.028	0.019
		3/4	0.011	0.010	0.023	0.019
		4/5	0.010	0.010	0.026	0.015
		5/6	0.010	0.007	0.020	0.013
		6/7	0.009	0.008	0.017	0.019
		7/8	0.012	0.011	0.017	0.017
		8/9	0.011	0.013	0.016	0.014
		9/10	0.013	0.013	0.018	0.013
		10/11	0.013	0.013	0.018	0.013
7	Control	2/3	0.010	0.009	0.020	0.017
		3/4	0.011	0.011	0.017	0.017
		4/5	0.012	0.008	0.016	0.016
		5/6	0.010	0.009	0.014	0.013
		6/7	0.009	0.008	0.012	0.012
		7/8	0.010	0.010	0.011	0.012
		8/9	0.011	0.008	0.011	0.011
		9/10	0.010	0.009	0.014	0.012
		10/11	0.011	0.009	0.018	0.018
7	Induced	2/3	0.012	0.013	0.009	0.017
		3/4	0.013	0.011	0.012	0.011
		4/5	0.009	0.009	0.008	0.011
		5/6	0.009	0.007	0.011	0.015
		6/7	0.008	0.011	0.008	0.011
		7/8	0.009	0.012	0.009	0.010
		8/9	0.010	0.012	0.011	0.011
		9/10	0.011	0.011	0.011	0.012
		10/11	0.017	0.018	0.013	0.011
14	Control	2/3	0.005	0.005	0.012	0.011
		3/4	0.011	0.010	0.009	0.009
		4/5	0.010	0.010	0.010	0.009
		5/6	0.010	0.009	0.008	0.008
		6/7	0.009	0.010	0.011	0.011
		7/8	0.009	0.009	0.010	0.009
		8/9	0.008	0.009	0.011	0.012
		9/10	0.009	0.008	0.012	0.013
		10/11	0.010	0.008	0.011	0.013

(*continued*)

Supplementary Table S4. (Continued)

Day	*Differentiation*	*Rank*	*Frozen pHPL vs. frozen FBS*		*Frozen FBS vs. fresh FBS*	
			100% efficiency	*Specific efficiency*	*100% efficiency*	*Specific efficiency*
14	Induced	2/3	0.011	0.009	0.007	0.010
		3/4	0.007	0.006	0.008	0.009
		4/5	0.007	0.007	0.007	0.009
		5/6	0.006	0.006	0.007	0.008
		6/7	0.007	0.006	0.006	0.006
		7/8	0.006	0.007	0.007	0.006
		8/9	0.007	0.009	0.009	0.009
		9/10	0.011	0.010	0.011	0.015
		10/11	0.017	0.015	0.018	0.019
21	Control	2/3	0.010	0.010	0.011	0.015
		3/4	0.011	0.007	0.015	0.014
		4/5	0.008	0.009	0.015	0.015
		5/6	0.010	0.009	0.013	0.013
		6/7	0.009	0.008	0.013	0.014
		7/8	0.009	0.009	0.012	0.014
		8/9	0.010	0.010	0.014	0.014
		9/10	0.012	0.010	0.015	0.018
		10/11	0.012	0.009	0.019	0.021
21	Induced	2/3	0.007	0.007	0.009	0.010
		3/4	0.006	0.006	0.009	0.008
		4/5	0.005	0.005	0.008	0.007
		5/6	0.005	0.004	0.007	0.008
		6/7	0.005	0.004	0.008	0.007
		7/8	0.005	0.005	0.009	0.010
		8/9	0.004	0.005	0.014	0.012
		9/10	0.008	0.007	0.014	0.019
		10/11	0.015	0.013	0.015	0.016
All		2/3	0.015	0.013	0.023	0.023
		3/4	0.016	0.016	0.018	0.017
		4/5	0.015	0.014	0.017	0.017
		5/6	0.012	0.012	0.013	0.014
		6/7	0.011	0.013	0.018	0.018
		7/8	0.013	0.012	0.019	0.018
		8/9	0.011	0.010	0.018	0.017
		9/10	0.010	0.010	0.016	0.016
		10/11	0.014	0.012	0.014	0.015
