# Supplementary material for: Reference Gene Expression in Adipose-Derived Stromal Cells Undergoing Adipogenic Differentiation
Source: Tissue Eng Part C Methods. 2019 Jun 17;25(6):353–66. doi: 10.1089/ten.tec.2019.0076 (PMC6589494; doi:10.1089/ten.tec.2019.0076)
Supplement: Supplemental data [file Supp_Fig2.pdf]

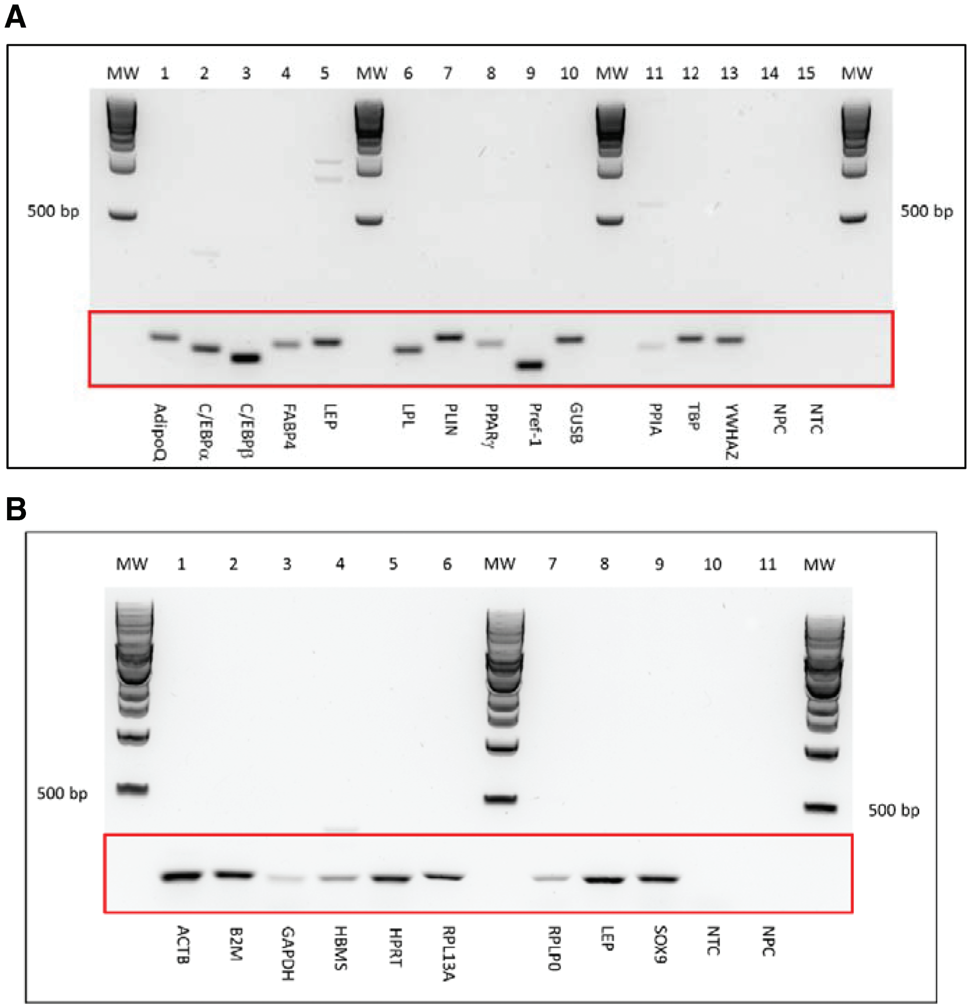

SUPPLEMENTARY FIG. S2. Confirmation of RT-qPCR primer specificity and amplicon length. Molecular weight (MW) marker (NEB 1Kb DNA Ladder, Inqaba Biotec, South Africa). Lanes of interest **(A)** Lanes: (11) *PPIA* (116 bp), (12) *TBP* (130 bp), (13) *YWHAZ* (126 bp), (14) NPC, and (15) no template control (NTC). **(B)** Lanes (1) *ACTB* (128 bp), (2) *B2M* (138 bp), (3) *GAPDH* (123 bp), (4) *HBMS* (130 bp), (5) *HPRT* (135 bp), (6) *RPL13A* (145 bp), (7) *RPLP0* (137 bp), (10) NTC, (11) NPC. Gel electrophoresis was performed using 2% (w/v) agarose (Lasec, Johannesburg, South Africa), and visualized using Gel red (Anatech, CA) on a ChemiDoc MP System (Bio-Rad, CA). Inverted images are shown. NPC, no primer control.
